# The Potential of Blockchain Technology for Health Information Exchange: Experimental Study From Patients’ Perspectives

**DOI:** 10.2196/14184

**Published:** 2019-06-20

**Authors:** Pouyan Esmaeilzadeh, Tala Mirzaei

**Affiliations:** 1 Department of Information Systems and Business Analytics College of Business Florida International University, Modesto A Maidique Campus Miami, FL United States

**Keywords:** health information exchange, patients, privacy, trust, risk, perception

## Abstract

**Background:**

Nowadays, a number of mechanisms and tools are being used by health care organizations and physicians to electronically exchange the personal health information of patients. The main objectives of different methods of health information exchange (HIE) are to reduce health care costs, minimize medical errors, and improve the coordination of interorganizational information exchange across health care entities. The main challenges associated with the common HIE systems are privacy concerns, security risks, low visibility of system transparency, and lack of patient control. Blockchain technology is likely to disrupt the current information exchange models utilized in the health care industry.

**Objective:**

Little is known about patients’ perceptions and attitudes toward the implementation of blockchain-enabled HIE networks, and it is still not clear if patients (as one of the main HIE stakeholders) are likely to opt in to the applications of this technology in HIE initiatives. Thus, this study aimed at exploring the core value of blockchain technology in the health care industry from health care consumers’ views.

**Methods:**

To recognize the potential applications of blockchain technology in health care practices, we designed 16 information exchange scenarios for controlled Web-based experiments. Overall, 2013 respondents participated in 16 Web-based experiments. Each experiment described an information exchange condition characterized by 4 exchange mechanisms (ie, direct, lookup, patient-centered, and blockchain), 2 types of health information (ie, sensitive vs nonsensitive), and 2 types of privacy policy (weak vs strong).

**Results:**

The findings show that there are significant differences in patients’ perceptions of various exchange mechanisms with regard to patient privacy concern, trust in competency and integrity, opt-in intention, and willingness to share information. Interestingly, participants hold a favorable attitude toward the implementation of blockchain-based exchange mechanisms for privacy protection, coordination, and information exchange purposes. This study proposed the potentials and limitations of a blockchain-based attempt in the HIE context.

**Conclusions:**

The results of this research should be of interest to both academics and practitioners. The findings propose potential limitations of a blockchain-based HIE that should be addressed by health care organizations to exchange personal health information in a secure and private manner. This study can contribute to the research in the blockchain area and enrich the literature on the use of blockchain in HIE efforts. Practitioners can also identify how to leverage the benefit of blockchain to promote HIE initiatives nationwide.

## Introduction

### Health Information Exchange Models

Individuals usually seek health care services from several providers who may practice in either affiliated or unaffiliated institutions. Accordingly, without a systematic connection among providers, patients’ medical information can become fragmented, outdated, and incomplete in health care organizations [1]. Health information exchange (HIE) is a data exchange mechanism that was introduced and prompted by the Health Information Technology for Economic and Clinical Health Act in 2009 to improve care coordination among health care providers and reduce medical errors [2]. HIE refers to the process of electronic transfer of patient health information and medical data among health care providers and institutions [3]. Interoperability associated with HIE initiatives requires electronic communication among organizations to ensure that patient medical records in one health care organization are seamlessly incorporated into another.

Different sharing mechanisms are being used by public and private health care organizations to facilitate information exchange initiatives [4]. Existing studies in HIE indicate that the following 3 exchange models are mainly applied by health care entities to electronically transmit patient health information: (1) direct, (2) query-based, and (3) patient-centered exchange [5]. In the direct model, a provider can share encrypted patient medical records with a known recipient [6]. This exchange model facilitates point-to-point data exchange in which the sender is aware of the recipient’s identity and patients’ medical records can be exchanged directly from one health care organization to another via widely adopted email protocols. Direct exchange initiatives, which are principally based on trust between providers, incorporate medical records into the recipient’s electronic health record (EHR) system or clinical inbox in a secure network governed by health care entities. The direct model is able to improve communication and coordination among health care organizations involved in providing treatments by securely exchanging identifiable information of patients.

The query-based models (lookup systems) grant health care providers the ability to find and request information on a patient from other providers. In this exchange mechanism, a central repository plays a critical role where electronic medical records are aggregated from multiple health care organizations’ EHR systems and will be stored in a hub [7]. Thus, the requesting health care organizations are able to use a lookup process to pull required information from the data storage pool [8]. The query-based model is mainly designed to create a mechanism to efficiently provide relevant, aggregated, and cross-organizational health records for care quality measurement and disease registries development.

The last model refers to a patient-centered exchange mechanism in which medical records related to episodes of care are transmitted from providers to patients. For instance, patients are able to view the laboratory results, radiology reports, progress notes, and medications that are uploaded on patient portals after each visit and share such records with other health care entities as required [9]. This exchange architecture is developed to enable patients to engage in their care process, manage their health information, and become a component of data-sharing efforts by considering a mediating role for them. Patients can leverage the patient-centered HIE models, which are designed and controlled by health care institutions, to reinforce their access and control over their own health records.

### Role of Patients in Health Information Exchange

Given the huge amount of information exchanged among health care organizations, patients would rely on HIEs to improve treatment process, enhance care coordination, and increase the quality of care before they actually experience the possible effects [10]. In this setting, risk can also arise because patients may be concerned that too much personal information is shared or erroneous health information is exchanged among health care providers through HIEs [11]. In the HIE context, patients may not directly share their health information through exchange mechanisms, and they are distant from care providers who actually use these systems. However, patients are recognized as an important beneficiary of HIE projects because their consent is required for sharing their health information [12]. Patients are also considered as a significant producer of health information and their attitudes toward HIE models may refrain them from sharing their personal information with HIE networks. If patients are not willing to share their personal health information, incomplete, outdated, or inaccurate patient information will be stored in shared records of HIEs [13]. Accordingly, HIE efforts will fail in providing health care providers with reliable, useful, and integrated health information. Previous studies highlight that to maximize the full value of HIEs, it is important to evaluate patients’ beliefs and perceptions about the widespread implementation of HIE networks [14]. Thus, public support is necessary for the long-term success and sustainability of HIE initiatives [8].

Different HIE models have attempted to clarify the process of electronic data sharing among health care entities. However, previous studies report that the general public is not completely aware of how health information is shared and used through the mainstream exchange mechanisms [15]. A number of studies highlight the importance of patient privacy and security concerns in the context of HIE implementation [16]. Patient concerns in medical practices include the volume of medical records collected and stored in health care organizations’ databases, the possibility of privacy violations (eg, unauthorized access or hacked personal data), secondary use of medical records (eg, datamining purposes), lack of control over data collection practices, lack of transparency associated with sharing efforts, and lack of visibility about how such information will be used [17]. Patients will hold a positive attitude toward HIE networks when their health records are collected, stored, and exchanged confidentially [18]. According to Wright et al [19], if a patient’s privacy and security needs are not met, he or she will become more likely to hide further health information from health care providers. Previous research indicates that patient decision to support HIE projects is a function of multiple factors such as type of information exchanged, privacy and security protections, and purpose behind the exchange [20]. Favorable attitude toward a HIE system is a result of a solid match between the HIE mechanisms and transparency, security, as well as privacy requirements [5].

### Blockchain in Health Care

Recent studies propose that blockchain is able to disrupt trusted business models mainly used in health care systems for information exchange purposes [21]. Considering the number of transactions (eg, information sharing) among health care entities and the expenses that hospitals experience in maintaining the HIE systems, the underlying blockchain technology of democratically sustained public ledgers of the records opens new and challenging opportunities for the health care industry. Blockchain can create an electronic context in which business transactions (such as information-sharing initiatives) between parties are conducted via a distributed community rather than a central authority or a single entity. This might essentially affect the transparency of the system and the role each entity plays [22]. Blockchain can also facilitate information exchange and coordination among health care entities and help patients become independent in the sharing of their medical records with providers. The mainstream HIE servers, depending on scale, are principally controlled by large corporations or health care institutions. This centralized control may raise privacy and security concerns because of abuses of power, which may result in secondary use of medical data, unauthorized access, and hacker attacks. Alternatively, the blockchain technology may promote a number of capabilities such as decentralization, security, privacy, breach resistance, and speed of certain features of the internet’s infrastructure.

A great deal of interest has been reflected by recent studies to analyze the effects of blockchain-distributed ledger technologies on health care practices, and most of them are conceptual research [23]. However, little quantitative work has been conducted to investigate the exposure of HIE to blockchain technology. Little is also known about patients’ attitudes toward the implementation of blockchain-enabled HIE networks, and it is still not clear if patients (as one of the key HIE stakeholders) are likely to opt in to the applications of this technology in HIE initiatives. Thus, more research is required to explore the core value of blockchain technology in the health care industry from health care consumers. Our work is among the first attempts to study the possible use of blockchain-based models in HIE from patients’ perspectives. The results of this research can extend the current understanding of blockchain technology by helping health care organizations, health care communities, and policy makers identify the potential benefits and risks of using this technology in health care practices. From a practical standpoint, this study can be useful for HIE policy makers to better examine the patients’ attitude toward the use of blockchain in HIEs, how it should be leveraged, and how patients can be impacted.

### Research Background

In this section, first the shortcomings and problems with traditional health exchanges are explained to better clarify the research gap. Then, we investigate blockchain-based HIE as a potential solution to the problems.

### Trust Issues in the Health Information Exchange Context

Trust plays a significant role in situations where there is a distance between consumers and vendors, such as in internet-dependent contexts [24]. HIE networks share individuals’ health information electronically with other care providers to improve care coordination and enhance patient safety. HIE initiatives utilize sharing mechanisms with which health information is mostly transmitted without a patient’s close supervision and control. Thus, patient’s trust in the HIE is the core in this setting where a great deal of security concerns and privacy risks may entail [5]. Trust in HIE can predict patients’ reactions to the implementation of HIE models because patients need to feel assured that the HIE networks will not compromise personal health information or misuse sensitive medical records [14]. Therefore, patients should trust HIE systems before they make an opt-in decision or disclose their personal health information.

Individual trust in HIE models can be a function of reliance on competence and integrity of sharing mechanisms [25]. Trust in HIE competence specifies the extent to which patients rely on technologically competent performance of the HIE to effectively disseminate health information between a wide variety of health organizations. Moreover, trust in HIE integrity refers to the belief that the agreement between the patients and HIE is reliable and honest. The lack of trust in HIE is mainly because of the distance imposed between patients and the actual users (health care organizations), lack of direct interactions between patients and HIE models, centralized control exerted by health care organizations, and the unfamiliar mechanisms used in the HIE system to share medical records electronically [26]. These characteristics create a setting that is more intangible than the traditional sharing methods (such as fax or mail). The mentioned reasons may make patient trust more critical in the settings where the 3 exchange models (ie, direct, query-based, and patient-mediated exchange) are mainly used.

### Privacy Concern and Privacy Policy

HIE initiatives are developed to provide interorganizational networks in which patients’ medical records are shared with a number of health care entities that are geographically scattered. When a networked-based technology (eg, HIE systems) deals with sharing sensitive information (such as health records), it is very likely that it exacerbates privacy concerns. Information privacy concerns may influence the validity and completeness of HIEs’ patient databases, which may result in wasteful investment, inaccurate treatments, erroneous care planning, and higher mortality rates [12]. To avoid such issues, HIE networks should assure patients that their medical records would be well protected during exchange transactions. Thus, privacy policies should be clearly presented by health care organizations to highlight how sensitive health information will be used inside/outside the organizations and what security means will be utilized to protect such data from unauthorized access and secondary use [27]. The risks of violated privacy, information misuse, or unauthorized disclosure highlight the importance of developing a transparent privacy statement before patient medical records are disclosed and shared.

Previous studies emphasize that patients are highly concerned about losing control over how the mainstream HIE systems handle their health information [28]. The concern is mostly because of a lack of transparency on the HIEs’ information practices and privacy policies. Privacy policies should be comprehensive and transparent enough to address all principles mentioned in the Health Insurance Portability and Accountability Act (HIPAA) [16]. The notice principle articulates what health information is collected and exchanged, what the purpose of data exchange is, how such information will be used internally, and whether patient data will be disclosed to third parties. The choice principle delineates the consent process and permission requirements. This dimension provides the choice to patients to put limits on providers for the exchange of health data. It also provides patients with the options to disclose such records to other third-party entities (eg, voluntary data disclosure for research purposes). The access principle entails granting the right to patients to obtain, review, and amend their personal information to ensure data accuracy and completeness. The security principle implies the adoption of reasonable measures and technical security steps to protect health information from unauthorized access, improper use, loss, unapproved alteration, or unanticipated disclosure during data exchange processes. The retention principle clarifies the acceptable duration of keeping and analysis of shared health information by health care providers for health care purposes. This dimension articulates the reasonable steps to permanently delete shared personal data if it is no longer required for the consented purpose. Finally, the enforcement principle highlights self-regulation such as privacy seals to protect information privacy by informing the public whether the exchange procedures correspond to the legal requirements [29]. Thus, highly transparent principles of privacy policies are able to demonstrate how safe, reliable, and dependent HIE networks are to reduce patients’ concerns for information privacy.

### Blockchain-Based Health Information Exchange as an Alternative

New ways of conducting business and operating economic activities are emerging through blockchain technology. Using dynamic shared ledgers, blockchain is able to facilitate recording business transactions between parties involved. Moreover, based on a peer-to-peer network of nodes, blockchain can also remove the need for intermediaries’ interactions and direct control by third parties in running a business. According to Crosby et al [30], the underlying features of blockchain make it to be considered as a disruptive technology that has potentials to fundamentally change current business models. Most studies in the blockchain domain have investigated cryptocurrency for its technical properties [31]. However, blockchain technology has broader and deeper applications beyond cybercurrencies and can be used for other purposes than financial transactions. As the interest in this technology has been rising, blockchain is attracting a great deal of attention and investment from numerous projects in different sections [32]. Blockchain technology is transforming several industries, such as banking, electronic governance, electronic commerce (e-commerce), legal contracts, automation, logistics, and health care [33]. Owing to its underpinning technology, one of the most conceivable applications of blockchain is in establishing coordination and managing communication between networked companies (such as hospitals). In a networked business model, all the involved companies are required to uninterruptedly communicate and constantly update their supply chain components to track the latest status of orders, processes, and transactions.

Blockchain may also contribute to other organizational initiatives such as information exchange across affiliated/unaffiliated health care entities (ie, all parties involved in the health care process, such as physicians, hospitals, and clinics). It has been proposed that blockchain-based sharing models, which use immutability and built-in autonomy features of the blockchain, are able to efficiently track records of access to sensitive medical data stored in the cloud [34]. According to Xia et al [35], health care organizations can take advantage of the access control framework that is based on blockchain to facilitate and expedite medical data sharing with other institutions. This technology provides secure cryptographic techniques to strongly control the access to patient medical records stored and processed on cloud platforms. Relying on the robust security platform, the system can detect and validate users that have access to sensitive medical records and keep track of all sharing activities.

The technology behind blockchain enables anonymous/pseudonymous actors in sharing initiatives, especially in a cross-border setting such as HIE. Blockchain can also resolve technical issues such as security and scalability as it operates based on a peer-to-peer network with no central authority, administrator, or a firm controlling the transactions. This decentralized network prevents a single point of failure and a security breach [36]. Moreover, cryptographic protocol used by blockchain technology provides communications security over a computer network. Using smart contracts embedded in blockchain technology, health care institutions can tap into automated execution of business interactions to notably decrease the need for majority of office operations in the sharing process.

**Table 1:** Descriptions of health information exchange models.

HIE^a^ model	Description	Reference
Direct exchange	Point-to-point information exchange in which a physician is able to share medical information with a known recipient over a secure network	Williams et al [6]
Query-based exchange	A single data repository that enables health care providers to share patient medical data with a centralized data warehouse. It also allows health care organizations to search for the required health information	Campion et al [5]
Patient-mediated exchange	This HIE model gives patients the ability to aggregate and manage their health information on the internet. Thus, patients can help share information between providers to track and monitor their own health	Rudin et al [43]
Blockchain-enabled exchange	A decentralized and trustless HIE model in which each block contains an episode of care and each node operates independently while following the sharing protocols. This model synthesizes medical data from patient-centered management tools and the EHR^b^ systems to provide access only to authorized stakeholders through secure transactions	Jiang et al [44]

^a^HIE: health information exchange.

^b^EHR: electronic health record.

Blockchain technology is considered as a trustless distributed ledger to collect, store, share, analyze, and validate medical data exchange among different stakeholders (such as health care organizations, providers, and patients) [37]. Therefore, one of the most promising applications of blockchain in the health care domain is in health data transmissions between patients, providers, hospitals, and relevant entities [38]. Blockchain technology has been suggested as an underpinning infrastructure for HIE to improve medical data storage, information exchange, and medical record management [39]. Recent studies also propose adoption of blockchain-based data-sharing networks to analyze secondary medical data for biomedical research purposes [21]. Another stream of research focuses on the use of blockchain to store patient-centered outcomes [40] and patient consent data [41]. Several companies, such as Deloitte [42], Accenture [41], and Guardtime [34], have initiated adoption of blockchain-based systems to store, manage, and exchange patient care. Therefore, consistent with previous research, blockchain technology is able to contribute to the health care industry and HIE efforts. In summary, the main characteristics of the 4 HIE models examined in this study are described in Table 1.

## Methods

### Experiment Design

**Figure 1:**
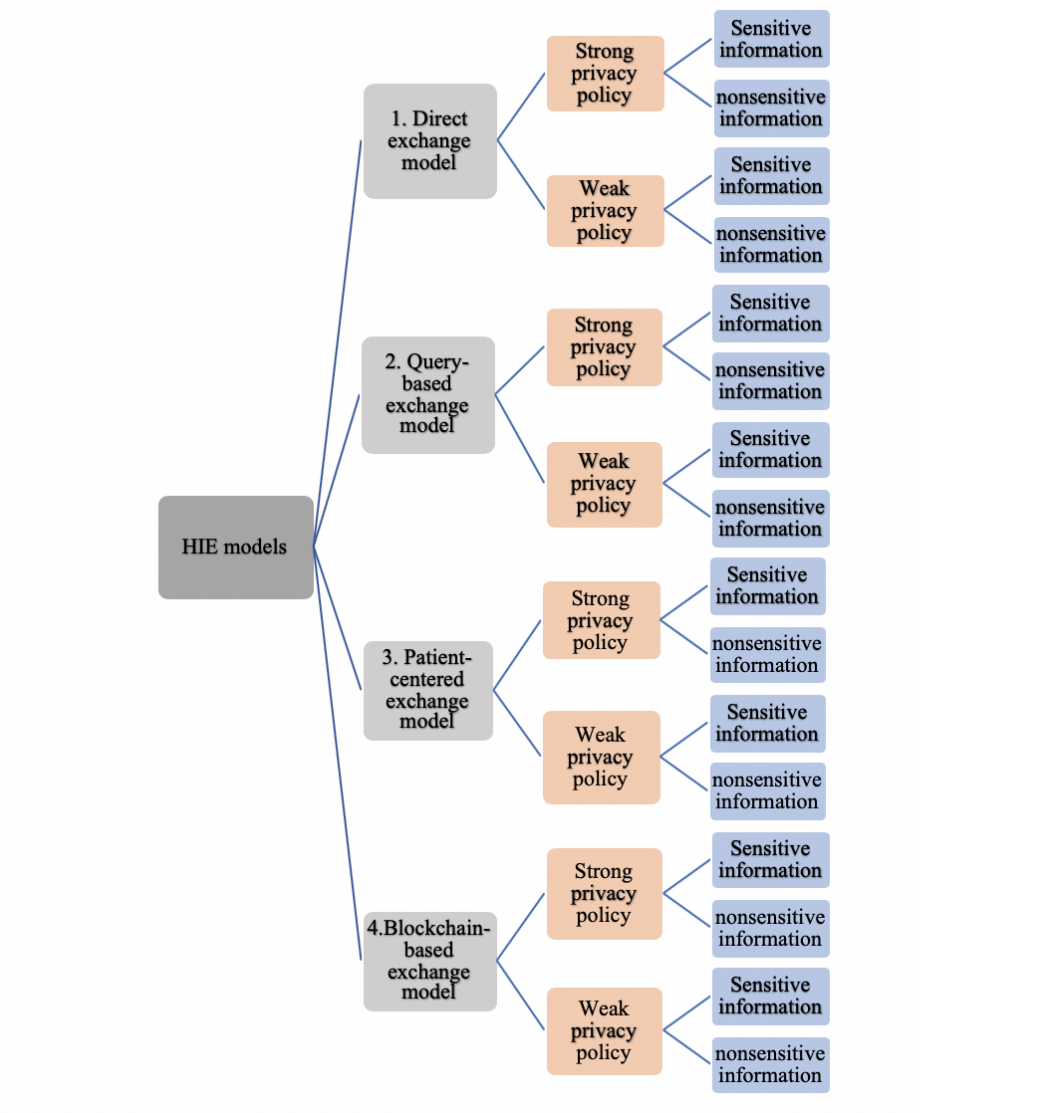
Sixteen scenarios.

We designed 16 scenarios to analyze health care consumers’ perceptions about the potentials and risks associated with the implementation of 4 possible HIE models (ie, direct, query based, patient centered, and blockchain based) built upon different architectures. The architectures of the 4 HIE models are different based on 2 factors: (1) transparency of privacy policy and (2) sensitivity of health information. In this study, we defined 2 extremes to examine the transparency of privacy policy used by the HIE models: strong versus weak. Moreover, we divided health information that could be exchanged through the HIE models into 2 types: sensitive versus nonsensitive. Figure 1 illustrates the 16 scenarios resulting from 4 HIE models, 2 types of privacy policy, and 2 types of health information.

Each scenario pertains to a separate experiment. Therefore, we conducted 16 separate experiments. As a between-subject experiment is a better choice than a within-subject experiment for attitude formation [45], in this study, we used between-subject experiments in which participants are randomly exposed to only 1 experiment. The total minimum sample required is 100 per experiment considering alpha=.05 and power beta=.95. As there are 6 main outcome variables in this study with 30 measures, we used minimum 120 respondents per experiment to reduce possible sampling errors. Table 2 shows the experimental design used in this study.

### Question Development

Each experiment included 8 sections: experiment scenario, health information privacy concerns, opt-in intention measures, trust in competency of HIE technology, trust in integrity of exchange transactions, willingness to share information, perceived benefits of HIE, and finally, demographics as well as technology experience questions. In the scenario section, a hypothetical situation was clearly described in which consumers were randomly exposed to a HIE model with particular characteristics. Each scenario envisions a situation in which a health care provider is explaining one of the exchange models defined in Table 2 and asking respondents to read the described privacy policy as well as type of health information that will be shared through the mentioned HIE model. For instance, in experiment 1, 128 respondents were randomly exposed to a direct exchange model with a strong privacy policy designed to exchange highly sensitive health information. To ensure that respondents completely understood the assigned treatments, we provided a detailed description of the given exchange technology and its features in terms of HIE model and architecture. We avoided any negative or positive connotations with the HIE models to resolve the possible bias that may arise from use of favorable/unfavorable terms. Then, subjects were asked to reflect their perceptions and opinions about the described exchange mechanism by answering a series of questions mainly developed according to previous research.

**Table 2:** Experimental design diagram.

HIE^a^ model	HIE architecture								
	Strong policy/sensitive information		Strong policy/nonsensitive information		Weak policy/sensitive information		Weak policy/nonsensitive information		
	Experiment #	n	Experiment #	n	Experiment #	n		Experiment #	n
Direct exchange model	1	128	5	123	9	128		13	132
Query-based exchange model	2	128	6	131	10	124		14	126
Patient-centered exchange model	3	122	7	126	11	127		15	125
Blockchain-based exchange model	4	120	8	125	12	126		16	122

^a^HIE: health information exchange.

This study drew on the existing literature to measure the constructs included in the model, and minor changes were made to the instrument to fit the HIE context. To design the scenarios, we adapted the 6 dimensions of privacy policy transparency reported by Chua et al [29] and Wu et al [46] to distinguish between a strong and weak privacy policy. The sensitivity of health care information was categorized based on the classification of sensitive information provided by National Committee on Vital and Health Statistics [47]. Respondents’ information privacy concern was measured based on their concern about the following items: collection, error, unauthorized access, and secondary use [48]. The scales used to measure trust in HIE technology’s competency and trust in the exchange mechanism’s integrity were adapted from a study conducted by Komiak and Benbasat [49]. Items measuring opt-in behavioral intention were adapted from previous research [50]. Items indicating willingness to disclose health information were adapted from the study by Zhang et al [51]. Items measuring perceived benefits were borrowed from factors suggested by previous studies [52,53]. All scales were measured on a 5-point Likert-type scale with 1 indicating *strongly disagree* and 5 indicating *strongly agree*. Finally, demographics and general technology experience questions were included at the end of the experiment (see Multimedia Appendix 1 for a description of the scenarios and questions).

We used the expert judgment approach to improve the content validity and completeness of our study. We sent the scenarios and questions to 5 professional health informatics practitioners and 3 blockchain experts. Then, the scenarios and questions were modified based on the experts’ suggestions to ensure that they were clear and easy to understand for the public. Before conducting the main study, we also conducted a pilot test with 86 students at a large Southeastern university in the United States. We provided an open-ended essay box at the end of the survey for the students to comment on the clarity of the scenarios and the questions. Furthermore, we followed up on the comments by conducting interviews with the students to understand any ambiguity in the scenario and the surveys. We revised the scenario and the surveys based on the comments from the students before final data collection. To ensure the reliability and validity of the instrument, the Cronbach alpha was computed for each construct (privacy concern alpha=.85, trust in competency alpha=.76, trust in integrity alpha=.91, opt-in intention alpha=.88, willingness to share information alpha=.90, and perceived benefit alpha=.93). All the Cronbach alpha values were above the cutoff point of .7, which indicated that the instrument was internally consistent [54].

### Recruitment and Participants

Data were collected in October 2018 using Amazon’s Mechanical Turk (MTurk) to obtain a representative group of subjects. MTurk is used by a number of studies as an acceptable means to collect individual-level data [55,56]. Research in different domains (especially psychological and social behavior) recruits respondents through MTurk to analyze the perceptions of samples that are more representative of the general workforce, including a wide range of ages, ethnicities, and work experiences [57]. We defined a location filter to collect data from the United States. The 16 experiments were posed to MTurk at the same time. We used a randomizer function to assign respondents randomly to the 16 scenarios to minimize the likelihood that 1 respondent could participate in more than 1 experiment. Moreover, a microcode was activated in the survey to keep individuals from taking each experiment more than once. Finally, all experiments were also double-checked using generated respondent identification and internet protocol address to ensure that the respondents were unique between experiments. The incentive for participation was a monetary reward. The range of average completion time for the 16 experimental groups was between 21:49 and 32:36 min that implied acceptable responses in terms of timing.

The 16 experiments obtained data from 2013 respondents, ranging between 120 and 132 participants each. We matched the respondents across the 16 groups to avoid any potential problem of individual differences between groups. Results of chi-square tests show that there were no significant differences among participants in all 16 groups, and they are very similar in terms of the demographic variables (see Multimedia Appendix 2 for results of chi-square tests). For instance, the distribution of data related to gender (*χ*^2^_15_=12.1; *P*=.66), age (*χ*^2^_75_=92.8; *P*=.08), health status (*χ*^2^_60_=49.9; *P*=.91), household income (*χ*^2^_60_=59.1; *P*=.51), race (*χ*^2^_60_=81.5; *P*=.06), education level (*χ*^2^_75_=76.1; *P*=.44), employment status (*χ*^2^_60_=69.1; *P*=.19), and computer experience (*χ*^2^_60_=51.7; *P*=.77) was notably similar across the 16 scenarios. Thus, we had enough evidence to assume that matched groups were used in this study (see Multimedia Appendix 2 for respondent characteristics across the 16 experiments).

## Results

### Analysis of Variance Test

We used IBM SPSS Statistics 24 to perform analysis of variance (ANOVA) to examine whether the 16 groups are significantly different by our main outcome variables: privacy concerns, opt-in intention, trust in competency, trust in integrity, willingness to share information, and perceived benefits. Before performing ANOVA analysis, we ran the Levene test to examine the homogeneity of variance, as this is one of the fundamental assumptions of 1-way ANOVA. The results do not show enough evidence to hold the assumption of homogeneity of variance for outcome variables. Therefore, we conduct Welch ANOVA that presents the most power and lowest type I error rate when data violate the assumption of homogeneity of variances [58]. Table 3 shows the descriptive statistics (mean score, SE, and Welch values) and the significance of each outcome variable.

**Table 3:** Descriptives and summary of analysis of variance results.

Outcome variable and scenario #		Mean		SE		Welch		*P* value	
**Privacy concern**						11.46		<.001	
	1		3.31		0.09				
	2		3.51		0.09				
	3		3.19		0.09				
	4		3.05		0.10				
	5		3.75		0.09				
	6		3.73		0.08				
	7		3.70		0.08				
	8		3.71		0.08				
	9		3.25		0.09				
	10		3.34		0.08				
	11		3.15		0.09				
	12		2.98		0.10				
	13		3.91		0.08				
	14		3.84		0.08				
	15		3.70		0.09				
	16		3.60		0.09				
**Trust in HIE^a^ competency**						5.64		<.001	
	1		3.46		0.07				
	2		3.19		0.09				
	3		3.40		0.07				
	4		3.35		0.07				
	5		3.16		0.09				
	6		3.23		0.07				
	7		3.09		0.07				
	8		3.14		0.09				
	9		3.38		0.08				
	10		3.34		0.07				
	11		3.32		0.08				
	12		3.52		0.07				
	13		3.00		0.09				
	14		2.95		0.09				
	15		3.00		0.09				
	16		2.97		0.08				
**Trust in HIE integrity**						8.40		<.001	
	1		3.47		0.08				
	2		3.10		0.09				
	3		3.30		0.08				
	4		3.29		0.08				
	5		2.95		0.09				
	6		3.04		0.08				
	7		2.96		0.08				
	8		2.93		0.09				
	9		3.35		0.08				
	10		3.36		0.07				
	11		3.34		0.07				
	12		3.52		0.07				
	13		2.90		0.09				
	14		2.91		0.08				
	15		2.90		0.10				
	16		2.85		0.09				
**Opt-in intention**						8.89		<.001	
	1		3.30		0.10				
	2		2.89		0.11				
	3		3.19		0.11				
	4		3.08		0.11				
	5		2.73		0.12				
	6		2.63		0.10				
	7		2.76		0.11				
	8		2.62		0.11				
	9		3.18		0.11				
	10		3.14		0.10				
	11		3.29		0.10				
	12		3.50		0.09				
	13		2.52		0.11				
	14		2.58		0.11				
	15		2.71		0.12				
	16		2.65		0.11				
**Willingness to share information**						6.67		<.001	
	1		3.35		0.11				
	2		2.95		0.11				
	3		3.19		0.10				
	4		3.19		0.11				
	5		2.81		0.11				
	6		2.70		0.10				
	7		2.82		0.11				
	8		2.68		0.11				
	9		3.21		0.11				
	10		3.13		0.10				
	11		3.32		0.10				
	12		3.36		0.10				
	13		2.56		0.11				
	14		2.70		0.11				
	15		2.81		0.12				
	16		2.73		0.11				
**Perceived benefits**						1.89		.02	
	1		3.84		0.08				
	2		3.61		0.09				
	3		3.68		0.08				
	4		3.59		0.08				
	5		3.59		0.09				
	6		3.55		0.07				
	7		3.59		0.07				
	8		3.47		0.08				
	9		3.76		0.08				
	10		3.62		0.08				
	11		3.75		0.09				
	12		3.76		0.08				
	13		3.52		0.08				
	14		3.68		0.07				
	15		3.63		0.08				
	16		3.45		0.08				

^a^HIE: health information exchange.

The results of this table demonstrate that there are significant differences across different scenarios at the *P*<.05 level for the 6 outcome variables: privacy concern Welch (15, 752.9)=11.455, *P*<.001; trust in HIE competency Welch (15, 753)=5.64, *P*<.001; trust in integrity Welch (15, 753)=8.39, *P*<.001; opt-in intention Welch (15, 752.99)=8.89, *P*<.001; willingness to share information Welch (15, 753.07)=6.67, *P*<.001; and perceived benefits Welch (15, 752.9)=1.88, *P*=.02. Therefore, comparisons indicate that the levels of privacy concerns associated with sharing activities, trust in HIE models’ competency, trust in integrity of sharing mechanisms, patients’ opt-in intention to HIE initiatives, patients’ willingness to disclose personal information, and perceived benefits of HIE networks significantly vary across the 4 HIE models, the 2 levels of health information sensitivity, and the 2 levels of privacy policy transparency. Furthermore, we conducted Games-Howell post hoc test, which is the multiple comparison procedure for means when variances and sample sizes are not equal, to identify which groups significantly differ from each other [59]. The following section describes the comparisons based on the 6 outcome variables used in this study.

### Privacy Concern

We compared respondents’ perception of privacy concerns associated with all 16 scenarios. For scenarios where a strong privacy policy is used to share sensitive health information, results reveal that privacy concern with blockchain technology is significantly lower than the direct exchange model (*t*=−1.97; *P*=.03) and the query-based model (*t*=−3.49; *P*<.001). Privacy concern for the patient-centered model is also significantly less than the query-based model (*t*=−2.64; *P*<.001). When comparing privacy concern between different HIE mechanisms for the scenarios where strong privacy policy is used to exchange nonsensitive information, we could not find any significant differences. In scenarios that use weak privacy policy for sharing sensitive information, we found that privacy concern with blockchain technology is significantly lower than the direct exchange model (*t*=−2.82; *P*<.001) and the query-based model (*t*=−2.06; *P*=.02). When respondents are exposed to scenarios with weak privacy concern for sharing nonsensitive health information, they express considerably lower privacy concern associated with blockchain technology compared with direct exchange (*t*=−2.65; *P*<.001) and query-based model (*t*=−2.05; *P*=.02). Overall, the results show that blockchain technology significantly reduces privacy concern among respondents compared with other HIE mechanisms regardless of the sensitivity of health information and strength of the privacy policy. Table 4 presents summary of significant results.

**Table 4:** Comparison of privacy concern across different health information exchange mechanisms.

Scenario		Health information exchange mechanism	*t*	*P* value
**Strong policy/sensitive information**				
	Blockchain technology	Direct exchange model	−1.97	.03^a^
	Blockchain technology	Query-based exchange model	−3.49	<.001^a^
	Patient-centered exchange model	Query-based exchange model	−2.64	<.001^a^
**Weak policy/sensitive information**				
	Blockchain technology	Direct exchange model	−2.82	<.001^a^
	Blockchain technology	Query-based exchange model	−2.06	.02^a^
**Weak policy/nonsensitive information**				
	Blockchain technology	Direct exchange model	−2.65	<.001^a^
	Blockchain technology	Query-based exchange model	−2.05	.02^a^
	Patient-centered exchange model	Query-based exchange model	−1.72	.04^a^

^a^The mean difference is significant at the .05 level.

### Trust in Health Information Exchange Competency

Next, we compared the participants’ responses to the level of trust in the capability of HIE mechanisms described in the sixteen 16 scenarios. According to Table 5, the results indicates that respondents who are exposed to strong privacy policies used to exchange sensitive information, express significantly more trust in the patient-centered exchange model (*t*=1.87; *P*=.03) and the direct exchange model (*t*=−2.39; *P*=.01) compared with the query-based model. We could not find significant differences in terms of trust in the competency of exchange technologies in other scenarios.

### Trust in Exchange Integrity

Regarding respondents’ level of trust in the integrity of the HIE mechanisms, the findings shown in Table 6 reveal that in the scenarios where sensitive information is shared with the help of strong privacy policies, there is a significant difference between blockchain versus query-based models (*t*=1.74; *P*=.04). In the same scenarios, our results show that trust in the integrity of the query-based model is significantly lower than that in the direct exchange model (*t*=−3.04; *P*=.001). There are no significant differences in terms of trust in the integrity and reliability of exchange mechanisms in other scenarios.

### Opt-In Intention

Furthermore, we compared the intention of respondents to opt-in toward a HIE mechanism that was presented to them by the given scenarios. In scenarios where sensitive information was shared based on strong privacy policies, we found significant differences between the query-based model versus all other HIE mechanisms. Table 7 shows that the query-based model is found to be the least favorite model for respondents. When a weak privacy policy is used to share sensitive information, participants are significantly more inclined to opt-in toward the blockchain exchange model versus all other HIE mechanisms. Moreover, in scenarios where nonsensitive information is exchanged under weak privacy policies, the blockchain technology is more favorable compared with the direct (*t*=2.57; *P*=.005) and query-based models (*t*=2.22; *P*=.01).

**Table 5:** Comparison of trust in health information exchange competency across different health information exchange mechanisms.

Scenario (strong policy/sensitive information)	Health information exchange mechanism	*t*	*P* value
Direct exchange model	Query-based exchange model	−2.39	.01^a^
Patient-centered exchange model	Query-based exchange model	1.87	.03^a^

^a^The mean difference is significant at the .05 level.

**Table 6:** Comparison of trust in exchange integrity across different health information exchange mechanisms.

Scenario (strong policy/sensitive information)	Health information exchange mechanism	*t*	*P* value
Blockchain technology	Query-based exchange model	1.74	.04^a^
Query-based exchange model	Direct exchange model	−3.04	<.001^a^

^a^The mean difference is significant at the .05 level.

**Table 7:** Comparison of opt-in intention toward different health information exchange mechanisms.

Scenario		Health information exchange mechanism	*t*	*P* value
**Strong policy/sensitive information**				
	Direct exchange model	Query-based exchange model	1.62	.04^a^
	Blockchain Technology	Query-based exchange model	2.71	<.001^a^
	Patient- centered exchange model	Query-based exchange model	1.93	.03^a^
**Weak policy/sensitive information**				
	Blockchain technology	Direct exchange model	2.95	.001^a^
	Blockchain technology	Query-based exchange model	2.63	.004^a^
	Blockchain Technology	Patient-centered exchange model	1.70	.04^a^
**Weak policy/nonsensitive information**				
	Blockchain technology	Direct exchange model	2.57	.005^a^
	Blockchain technology	Query-based exchange model	2.22	.01^a^

^a^The mean difference is significant at the .05 level.

### Willingness to Share Health Information

We further investigated whether respondents are willing to share their health information given the scenarios. In scenarios where sensitive information is shared under strong privacy policies, participants prefer blockchain technology significantly more than the query-based model (*t*=3.03; *P*=.001). Table 8 also shows that in the same scenarios, respondents express more willingness to share their information through the patient-centered model the than query-based model (*t*=2.01; *P*=.02). In scenarios where sensitive information is exchanged based on weak privacy policies, respondents exhibit significantly more willingness to share health information through blockchain technology compared with the direct model (*t*=5.07; *P*<.001), query-based model (*t*=5.61; *P*<.001), and patient-centered model (*t*=4.21; *P*<.001). In the same scenarios, we also found that respondents prefer the patient-centered model better than the query-based model (*t*=1.95; *P*=.03). Moreover, participants show more willingness toward blockchain technology for sharing nonsensitive information under weak privacy policies compared with the direct model (*t*=3.89; *P*<.001), query-based model (*t*=3.27; *P*<.001) and patient-centered model (*t*=2.001; *P*=.02). In the same scenarios, respondents also prefer the patient-centered model versus the direct model (*t*=2.09; *P*=.02) and the query-based-model (*t*=2.001; *P*=.02).

### Perceived Benefits

**Figure 2:**
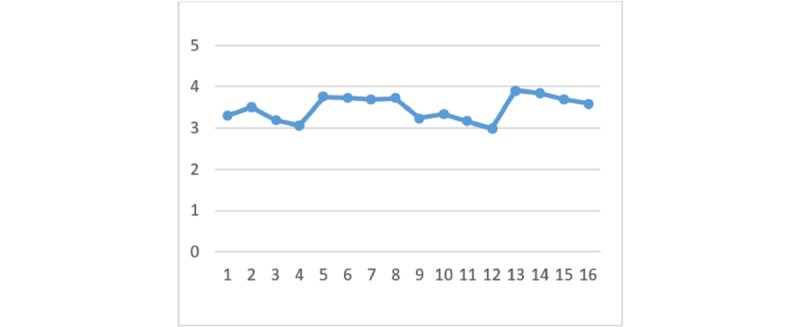
Differences in means of privacy concern.

**Figure 3:**
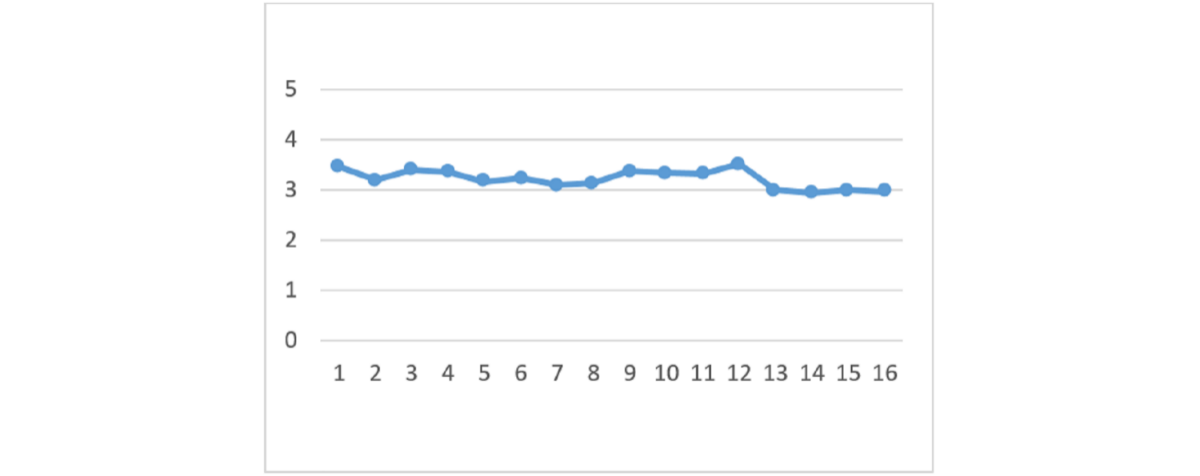
Differences in means of trust in health information exchange competency.

**Figure 4:**
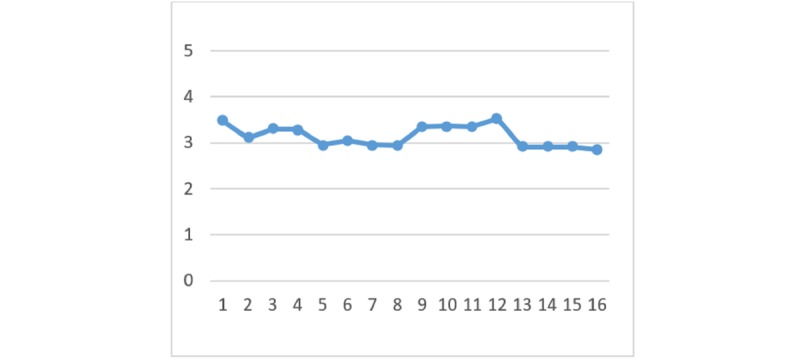
Differences in means of trust in exchange integrity.

**Figure 5:**
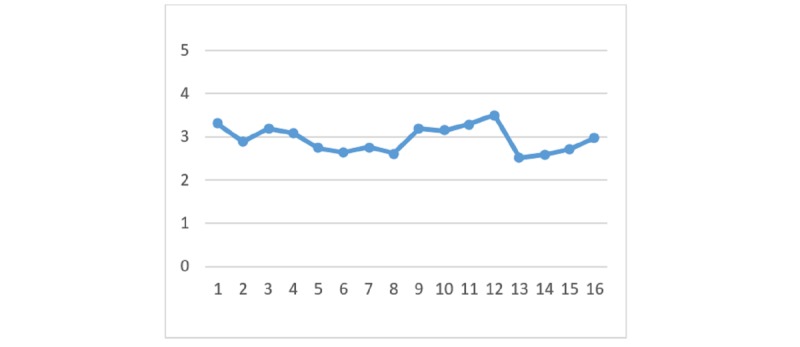
Differences in means of opt-in intention.

**Figure 6:**
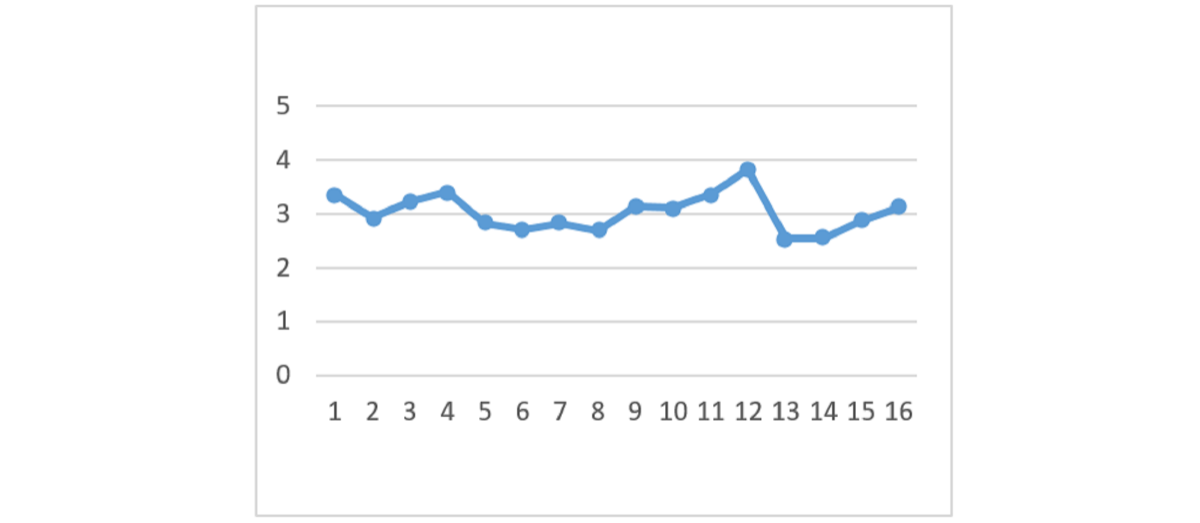
Differences in means of willingness to share information.

**Figure 7:**
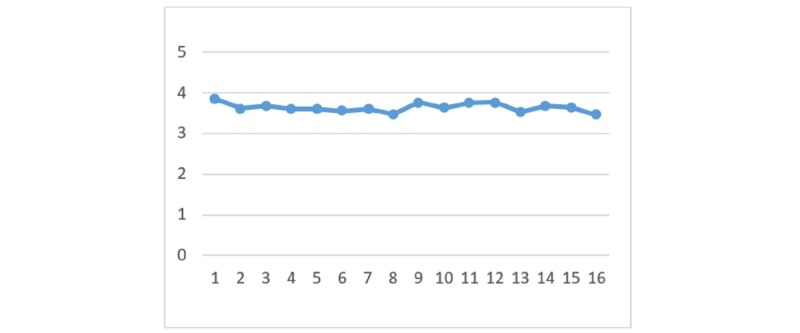
Differences in means of perceived benefits.

With regard to the perceived benefits of HIE, there are no significant differences between the 4 HIE mechanisms given the different types of privacy policy and information sensitivity. Figures 2 to 7 display the differences in the means of different scenarios for each outcome variable.

**Table 8:** Comparison of willingness to share information across different health information exchange mechanisms.

Scenario		Health information exchange mechanism	*t*	*P* value
**Strong policy/sensitive information**				
	Blockchain technology	Query-based exchange model	3.03	.001^a^
	Patient-centered exchange model	Query-based exchange model	2.01	.02^a^
**Weak policy/sensitive information**				
	Blockchain technology	Direct exchange model	5.07	<.001^a^
	Blockchain technology	Query-based exchange model	5.61	<.001^a^
	Blockchain technology	Patient-centered exchange model	4.21	<.001^a^
	Patient-centered exchange model	Query-based exchange model	1.95	.03^a^
**Weak policy/nonsensitive information**				
	Blockchain technology	Direct exchange model	3.89	<.001^a^
	Blockchain technology	Query-based exchange model	3.27	<.001^a^
	Blockchain technology	Patient-centered exchange model	2.001	.02^a^
	Patient-centered exchange model	Query-based exchange model	2.04	.02^a^
	Patient-centered exchange model	Direct exchange model	2.09	.02^a^

^a^The mean difference is significant at the .05 level.

## Discussion

### Theoretical Implications

This study has implications for researchers conducting studies in the HIE context. Our study is different from previous research by examining patients’ perspectives of 4 HIE models. This research is mainly designed to address how different models of HIE can affect patients’ attitude toward electronic data exchange between health care providers. To do so, we investigated whether levels of patients’ privacy concerns, perceived benefit of HIE, trust in HIE competency, trust in HIE integrity, willingness to share personal information, and opt-in intentions are different across multiple HIE models (ie, direct, query based, patient centered, and blockchain based). This study also contributes to the literature by providing new insights on how blockchain technology can be leveraged in the context of HIE and how patients may be affected.

#### Blockchain Technology

The content of the blockchain is information; thus, its use is adaptable in different business sectors. In line with the study by Liu and Tsyvinski [60], industries have different reactions to blockchain as they may benefit or become disrupted by this technology. Although there are several attempts among information systems (IS) scholars to recognize the applications of blockchain technology in different business contexts, significant theoretical contributions are still scarce, especially in the health care context. Economists, computer scientists, finance scholars, and IS scholars analyze blockchain technology and its adoption from various lenses. According to the existing blockchain literature, the majority of studies focus on its applications in financial transactions. This research domain can be divided into 5 main categories. One group of studies has focused on the potential use of cryptocurrency for illegal activities and has not examined the motives of mainstream users to adopt it for legitimate uses such as for e-commerce, information exchange, or money transfer [61]. The second group of studies has investigated cryptocurrency for its technical properties such as design science, cryptography, proof-of-work algorithm, or exchange rates perspectives [31]. The third category of research has called readers’ attention to analyze the differences between the technical, usability, and social characteristics of different forms of cryptocurrencies [62]. The fourth category of studies has attempted to distinguish cryptocurrency adopters from nonadopters based on either drivers or risks associated with its underlying technology [63]. The last category has used widely accepted adoption models (eg, technology acceptance model [TAM] and unified theory of acceptance and use of technology [UTAUT]) and mainly focused on the perceived usefulness and perceived ease of use in the context of cryptocurrency [64].

Blockchain-based sharing frameworks to facilitate the exchange of medical information between health care and research institutions are under development. These medical blockchain applications sufficiently control access to medical data stored and processed on cloud systems. They also offer secure cryptographic techniques to identify and authenticate users who have access to medical data to keep track of all exchange transactions [34]. Thus, sharing data for telemedicine and medical consultations in remote areas becomes more efficient. One of the main factors affecting the widespread adoption of blockchain-based HIE is social acceptance of this exchange mechanism. A patient may seek medical treatments and care planning from different health care organizations and providers. In a situation where patient-provider interactions are growing, a technology may be required to facilitate communications and medical records exchange without a centralized authority but relying on a distributed public ledger of all data exchange transactions. However, previous research on how patients would react to medical information sharing through blockchain is still in its nascent stage. In this study, we analyzed 6 outcome variables that need to be considered to measure the success and effectiveness of HIE models from patients’ views. This work is among the first studies to empirically examine the potential role of blockchain in the HIE context. The results imply the significance of blockchain-based technology for health care applications when compared with the mainstream HIE models (ie, direct, query based, and patient mediate exchange).

#### Privacy Concerns

With regard to the privacy concern, blockchain-enabled HIE models in different scenarios (when either sensitive or nonsensitive health care information is shared under a strong or weak privacy policy) to receive favorable evaluations from our respondents. This is consistent with the study by Abdulnabi et al [18], which indicates that more decentralized models that increase patients’ control over their medical data and exchange transactions will be more feasible and applicable approaches for HIE efforts. According to Vest and Gamm [3], using a centralized data repository in HIE initiatives has heightened privacy and security concerns for patients and created control issues for health care organizations. Moreover, an HIE model that uses blockchain technology to exchange sensitive information even under a weak privacy policy has more information privacy advantages from patients’ perspectives compared with all other exchange models. Therefore, the findings show that the public considers blockchain as a more secure exchange model to share highly sensitive medical records regardless of privacy policy transparency. However, it can be discussed that open transparency of information (especially for sensitive records) during transfer can be obscure for consumers and should be addressed. This is in line with previous studies that indicate that blockchain is characterized as a decentralized, distributed, immutable, and transparent technology that can be used as permission-less or permissioned networks [23]. In the permission-less blockchain networks, any users can involve and participate without being authorized, and in the permissioned networks, only authorized users or organizations can participate. Owing to the overall sensitivity of health care information, stricter policy guidelines, and high compliance requirements in the context of HIE, the permissioned blockchain-based network would be a more secure option to enable electronic exchange of medical data with providers participating in other settings. This point is also highlighted by previous research indicating that although current blockchain technology underlying cryptocurrency is not fully anonymous, transaction anonymization for legitimate purposes (such as health care services) is desirable [65]. For example, in HIE networks, confidential health information should be handled with optimum security protocols.

#### Trust in Health Information Exchange Models

Sharing sensitive health information through a technology that is used by health care providers requires a new lens for understanding patients’ trust in HIE technology. Regarding the trust in HIE competency and exchange integrity, a blockchain model even with no strong privacy policy is found as the most trustable model than other exchange mechanisms for sharing highly sensitive information. Consistent with this result, the public may believe that blockchain HIE has the necessary characteristics, technological capability, and features to be relied upon, regardless of presenting a comprehensive and transparent privacy policy for transmitting sensitive medical records (such as genetic information, mental health information, sexual health diseases, substance abuse, and addiction). Thus, blockchain HIE may heighten patients’ cognitive dependence on HIE integrity and competence and win the trust of patients to exchange sensitive health-related information. This is consistent with previous studies that blockchain can be used as a reliable technology to share both highly sensitive medical data and less sensitive information such as current health statues (eg, fitness, diet, diseases, and treatments) or past medical/health information (eg, list of vaccinations and medications used) [35].

Consistent with previous studies, in the process of forming trust in technology (as an impersonal entity), consumers’ awareness of the unknown should be resolved [66]. Previous research indicates that the public awareness about HIE mechanisms, functions, integrity, and security safeguards needs to be raised [2,26]. For example, one area could be the differences between the open transparency of information in cryptocurrency and blockchain-based HIE. The transparency of information in cryptocurrency means that all nodes in the network have the right to access the whole information related to financial transactions. However, this feature is not desirable for transmitting highly sensitive health information. To implement blockchain exchange methods to share sensitive health data across providers, it is required to develop security features (eg, confidentiality, availability, and integrity), which is considered as one of the main aspects of blockchain technology. A blockchain-based HIE system is a decentralized framework where all medical records are confidential and the availability of such information does not rely on any third parties (eg, hospitals or providers). Furthermore, data integrity can be ensured because this form of HIE uses a distributed file system where participants in exchange activities will keep copies of all files, including the shared health information. Moreover, they agree to share, change, and update medical data by permission requests and consent processes. Therefore, the rational expectations about the HIE’s ability to fulfil its obligations (cognitive trust in competence) and the rational reasons associated with the reliability of the HIE principles (cognitive trust in integrity) can be increased through raised awareness about the use of various types of blockchain innovations such as smart contract applications and permissioned networks.

#### Opt-In Intentions to Health Information Exchanges and Willingness to Share Information

Patients are considered as one of the most important stakeholders of any HIE efforts as the widespread implementation of HIE projects will not be feasible without their positive beliefs and attitudes toward the exchange models, their opt-in intentions to HIE initiatives, and their willingness to share health information [67]. The existing theories of information technology (IT) adoption (such as TAM and UTAUT) focus on users’ intention to accept and use a technology [68]. However, in the HIE context, patients are not the main users. Patients are the beneficiaries of HIE initiatives, but they are not the final users. The users are health care professionals (ie, physicians and nurses), and the decision to adopt HIE is made at the practice/hospital level. However, it is critical to study whether patients will choose to opt in to HIE systems or they will not support such initiatives by hiding their personal health information. The results show that participants are most likely to opt in to blockchain HIE as a reliable technology to be used by health care entities to disseminate highly sensitive information even in the absence of a strong privacy notice. This finding is consistent with previous research highlighting that patients are more favorably disposed toward decentralized HIE models versus centralized exchange systems [69]. Furthermore, respondents are most willing to disclose sensitive health information to health care organizations, with the knowledge that such information may be exposed to other providers through a blockchain-based HIE even when privacy policy is not completely transparent.

The results manifest that with the current blockchain technology, patients may not feel skeptical about relying on blockchain-enabled HIE to manage the exchange of their highly sensitive information among a wide range of providers. This finding also emphasizes the importance of raising patient awareness of how the consent process and permissioned HIE networks operate in practice. Moreover, more efforts are required to improve the legal image of blockchain technology in health care to enable at-scale interoperability for information exchange, patient tracking, identity assurance, as well as validation among health care institutions and between patients and their providers [41]. Our findings also propose possible direct relationships of trust in blockchain HIE with patients’ opt-in intentions and their willingness to disclose health information. Thus, a high level of trust in blockchain competence and integrity may encourage patients to opt in to this technology and disclose their sensitive health information when visiting a physician participating in a blockchain-based HIE network.

#### Perceived Benefits of Health Information Exchange

Pertaining to the perceived benefits of HIE, there is no significant difference across the scenarios. The 4 HIE models, regardless of different architectures (privacy policy and data sensitivity), receive the same level of benefits from patients. This means that although information privacy concerns can cause significant differences, all the exchange models are perceived to deliver comparable values. Thus, the instances that privacy policy dimensions are not stated transparently or conditions that highly sensitive medical data are likely to be shared will not significantly affect the core values expected from the HIE models. This is in line with previous studies that multiple exchange mechanisms may be used to fulfil different health care needs but the main purpose of all HIE models is to support care coordination, reduce health care costs, and improve patient safety [70]. Thus, patients may believe that regardless of what exchange model will be used for sharing information, HIE initiatives are generally able to improve communication among health care providers, reduce delays in care delivery, and advance quality of care planning.

### Practical Implications

#### Patient Awareness About Blockchain-Based Health Information Exchange

There are also a number of important practical implications derived from this study. First, the findings suggest the importance of educating consumers about the use of blockchain technology in HIE mechanisms and sharing procedures. For instance, national educational programs, health conferences, and webinars that are easily accessible to a wide range of people can be administered to clearly publicize the key goals and advantages of blockchain-based HIE efforts. Educational forums available on official health websites, Web-based tutorials accessible on patient portals or Web-based health communities, and computerized help programs can be used by health care organizations to improve the transparency of blockchain applications in HIEs, broadcast their expected benefits, and increase public awareness and patient familiarity with this exchange mechanisms.

Second, regarding the importance of information privacy in blockchain-based HIE, health care providers should consider using tactics to increase the transparency and completeness of privacy policy and invest considerable effort in developing campaigns that leverage the power of blockchain image and reputation in health care. HIE policy makers should establish a broad marketing strategy to enhance patients’ perceptions about the accountability and accuracy of privacy policies, which can foster patients’ opt-in intention toward blockchain-enabled HIE services. Research implications suggest that HIE initiatives’ managers should consider maximizing the transparency of privacy policy dimensions to encourage consumers to read the privacy policy statements when data are subject to be exchanged through the blockchain networks.

Third, lack of public awareness about the blockchain-based HIE model as well as the components of its privacy statement may impede the progress of sharing information between providers because of the lack of patients’ support for HIE. This study suggests that both physicians and health care organizations (such as hospitals) can directly play an important role in persuading patients to give consent to sharing medical records using blockchain-enabled HIE. Physicians’ role may be more effective because they have face-to-face encounters with patients and during consultations, they can enlighten the patients about the benefits of using blockchain in HIEs, and how they could be impacted. For instance, health care professionals can explain how HIE, which is enabled by blockchain can help physicians detect diseases faster, coordinate treatments with other providers, and finally, improve patient safety. Hospitals can also influence how patients shape opt-in decisions toward blockchain-based HIE by educating them using brochures, leaflets, diagrams, and fact sheets that are comprehensible for an average person. These efforts should be able to clearly highlight why health information is shared, what types of information can be exchanged, how such information is shared from one point to another, what exchange mechanisms are used, who can access the medical data, what security safeguards will protect their records, and how often the transmission takes place.

#### Potential Benefits of Blockchain-Based Health Information Exchange

Relying on the key findings as well as characteristics and features of blockchain, the main benefits of using blockchain for improving medical record sharing among health care organizations are discussed in the following section. Decentralized management of the blockchain technology can notably contribute to HIE by providing patient-managed health care records. In these platforms, patients are considered as the owner of their medical records and are able to efficiently control access to such information [37]. This aspect can also help patients reduce all possible barriers associated with obtaining copies of their medical information and potential risks related to sharing them with other health care organizations. In a blockchain-based HIE, each block can contain an episode of care and each node operates independently while following the sharing protocols. Blockchain has the potential to become an electronic health information pool by synthesizing medical data from patient-centered management tools and the EHR systems to provide access only to authorized stakeholders (such as patients and providers). The peer-to-peer architecture of blockchain also enables health care institutions to keep control of their own IT resources and collaborate with other organizations to enhance information sharing initiatives without ceding control [71]. Thus, incorporating blockchain into HIE is appropriate for health care providers/organizations that seek to cooperate with each other with no centralized management intermediary. On the contrary, most of HIE mechanisms (eg, direct model or lookup networks) are centrally managed.

The immutable audit trail is another characteristic of blockchain technology that is likely to contribute to HIE. On the basis of this aspect, patient health information is not changeable in any steps of the data-sharing initiatives. Thus, the medical records that are stored in the private blockchain cloud cannot be altered, manipulated, or removed by any entities participating in HIE initiatives such as health care providers and organizations [37]. Furthermore, patient medical records that are generated and shared with health care providers through a blockchain-based sharing platform are trackable and timestamped.

Managing patient consent records during data-sharing processes can be improved by the data provenance of blockchain technology. This aspect can help the owner of medical records to change the ownership or give permission to other entities to view, process, and share such information using the cryptographic protocols. Moreover, patients or providers can trace the source of data and verify legitimacy as well as accuracy of records to be used for exchange purposes. Thus, using blockchain-based HIE, the source of medical records is detected and any ownership transfer in each block will be transparent and available to everyone involved in the data-sharing efforts.

Blockchain is built upon distributed technology that does not suffer from a single point of failure. Relying on this feature, patient health information can be collected, stored, and shared on a decentralized network, where there is no central institution that could be hacked or compromised. This robustness feature has the potential to decrease the risk of patient recordkeeping as medical data cannot be faked or manipulated. Moreover, one of the main threats related to the mainstream HIE models is unavailability of patient data when incomplete or inaccurate patient information is stored in shared records [12]. This issue can be resolved by blockchain technology as each node in the network has a copy of historical medical records and is able to continuously update such data. This characteristic may guarantee that the electronic medical records of patients are continuously available in real time [72]. Real-time access to patient data is one of the main promises of HIE efforts that enable providers to advance care coordination, detect epidemics rapidly, and improve care delivery in emergency situations [73].

All information exchange initiatives in the United States health care industry (such as HIE projects) fall under HIPAA security rules [25]. Under HIPAA, security policies and procedures should be implemented to prevent, detect, and correct security violations [74]. For example, a thorough analysis of the potential risks and vulnerabilities to the confidentiality, integrity, and availability of electronic health information held by the covered entity should be conducted before exchanging any information. Moreover, procedures for the authorization and supervision of members who use electronic protected health information should be clarified [16]. For instance, security procedures should determine whether the access of a health care organization to electronic health information is appropriate or should be terminated. On the basis of HIPAA guidelines, procedures are implemented to verify that a physician or entity seeking access to electronic health information is the one claimed [75]. For instance, technical security measures and encryption mechanisms are implemented to guard against unauthorized access to electronic health information that is being transmitted over an electronic communications network (such as HIE).

A number of studies have argued that privacy and security concerns are identified as the most pressing barriers to widespread consumer participation in the implementation of mainstream HIE models [53]. Privacy policies of HIE efforts should be comprehensive and transparent enough to address all the principles mentioned in HIPAA [16]. One of the main advantages of blockchain technology that can be utilized by HIE models is improving safety, integrity, and confidentiality of patient health information using cryptographic algorithms and consent recording systems. Episodes of medical care can be stored in blocks and only decrypted for exchange purposes with the patient’s private key. Even if the distributed network is breached by a malicious entity, with current technology means, it is extremely unlikely that patient data can be illegally accessed by unauthorized parties. Therefore, blockchain-enabled sharing platforms have the potential to connect a vast number of patients, health care providers, and health care organizations to exchange variety of medical records while information privacy and security are protected.

#### Plausible Challenges of Blockchain-Based Health Information Exchange

The objective of this study was not to propose blockchain as the most advantageous method of information exchange in the health care industry. Results of our research indicate that there are still a number of criticisms attributed to blockchain-based solutions in the health care area. In this section, the main shortcomings of blockchain are highlighted to imply that current blockchain solutions need some necessary modifications to be implemented in the HIE context.

Although blockchain is an appropriate means to facilitate interoperability, current studies have also emphasized that the open transparency of data during exchange transactions is not desirable in health care applications [41]. In the HIE context, identifiable information of patients is highly sensitive. The key objective of HIPAA compliance is that information exchange must be protected against a confidentiality breach. The end-to-end workflow of a blockchain-based HIE (ie, entering, processing, and delivering of health data) must be HIPAA compliant. Any personal health information accessed by the blockchain-enabled HIE must be encrypted and securely managed by parties interacting with this HIE model. A blockchain-based HIE should not make all personal information publicly available so it can securely store and manage sensitive data. Blockchain-enabled HIE should ensure the anonymity of each identity and transaction using unique authentication protocol (data protection methods such as tokenization or masking) [76]. Thus, each data exchange performed by a user should not be linked to the user and the ownership of the key should remain anonymous. Moreover, privacy policies designed for blockchain-based HIE can provide different levels of data access and, if required, time-limited access. Another way to alleviate the open transparency issue is encrypting sensitive health records on the network of blockchain-based HIE [77]. Recent studies also propose that sensitive medical records can be stored off-blockchain network and only encrypted links and permission information should be exchanged on network [38]. According to Ekblaw et al [78], data exchange protocols can be automated using smart contracts to attenuate this risk.

Another potential challenge with the adoption of blockchain in HIE networks is the speed of transactions. Depending on the authentication and verification protocol used in blockchain, data exchange processes could be time consuming. This could challenge the real-time communication, coordination, and data sharing among health care providers, which is critical in many health care situations [23]. According to Linn and Koo [72], ongoing verified exchange transactions can only be stored in blocks instead of the complete past medical histories. Another plausible solution is to implement blockchain-based platforms that provide higher transaction speed compared with the Bitcoin network [79].

The risk of a 51% attack has been considered as an important threat to blockchain networks [80]. This attack, which occurs when the whole network is controlled by attackers or malicious nodes, could critically threaten the security of HIE platforms. HIE can adopt permissioned blockchain networks in which malicious nodes are not able to randomly contribute to the network, and in turn, the risk of a 51% attack could be minimized. For instance, implementation of a virtual private network in which medical records are stored and exchanged on private cloud resources complied with HIPAA can notably mitigate this risk [81].

Finally, it should be mentioned that the spread of the blockchain-based framework in health care practices might be challenging, particularly in developing countries that do not have adequate technical infrastructure and social support. Moreover, the long-term success of blockchain-based HIE needs favorable attitude and active participation of all stakeholders (such as physicians, health care organizations, and patients). According to Dixon et al [82], HIE projects may become ineffective and disabled because of a lack of participation and support from HIE stakeholders. With respect to patients, there is a need to increase public awareness about blockchain technology. For instance, national educational programs such as educational videos or webinars can be used by health care providers to convey key information about blockchain-based HIE and how it facilitates the sharing of medical data securely with and between health care providers. Patients should be educated on the aspects of blockchain to realize how the technology is able to exchange sensitive medical data securely, improve confidentiality of all sharing activities, enable patients to track who can access episode-of-care data, and increase patient control over their medical records.

### Limitations and Future Research

Similar to other studies, our research has some limitations that call for additional work. We began this study by reflecting on patients’ perceptions about the implementation of 4 HIE models. Researchers coming from a different starting point could contribute to this research stream in different ways. We raise this point, not to defend our view or to deflect criticism, but simply to clarify the scope of our paper and motivate future research that takes different perspectives or assumptions. For instance, future work can examine health care professionals’ perspectives or investigate health care organizations’ requirements and limitations on the implementation of blockchain-based HIE alternatives. This study is mainly designed based on the hypothetical scenarios that clearly define the use of 4 HIE models under different circumstances (ie, privacy policy and type of information). Relying on existing literature, expert judgment approach, and pilot testing, we provided clear definitions by articulating the HIE models, privacy policy, and data sensitivity to reduce possible ambiguity. However, as HIE still is a relatively new technology, there was a small chance that some respondents did not comprehend the scenarios completely. Thus, we suggest that further studies use samples who have experience with the HIE models.

Consistent with the results of this study, further research can also develop and empirically test a causal model using the outcome variables proposed by this study to predict the success of blockchain in HIE initiatives from consumers, health care professionals, and hospital managers’ perspectives. Health care industry is considered as a highly regulated environment. Future studies can extend this work by identifying approaches to address governance conflicts arising from the technology being used in the health care context. It can also be of interest for future research to investigate the role of regulatory bodies in keeping control, on the one hand, and having systems that run on their own, on the other. In this study, we discussed the key risks involved along with several plausible solutions related to the adoption of blockchain technology in the HIE context. Future research is required to shed more light on the design and implementation of blockchain-enabled HIE applications. Finally, this study provides a footstone for further theoretical development and practical investigation. For instance, future work can study the return on investment and cost impact to health care delivery as a result of a blockchain-enabled HIE implementation. Moreover, the legal and policy implications/requirements can be addressed by further research.

### Conclusions

Blockchain is considered as one of the most important technologies that can be applied in many sectors in the future. One of the most interesting cases of blockchain technology application is in health care domains. Research on the use of blockchain technology in the health care context is still in its early stages, and its widespread adoption needs further efforts. This work uses an experimental approach to better articulate the prospective application of blockchain technology in creating an infrastructure for sharing medical records. The findings indicate that blockchain technology has a great potential to be integrated in existing HIE architectures to improve system transparency, patient consent tracking, and privacy protection of information exchange initiatives. Blockchain-based HIE is able to provide a platform for data exchange that does not need a centralized authority to operate. This aspect promotes a protocol supporting a network-based communication between patients and physicians and a well-organized coordination among health care organizations to accurately diagnose diseases, provide timely treatments, and improve patient safety. According to the results of this study, patients perceive that blockchain technology can be a reliable replacement for current exchange models, which are mainly managed by mainstream bureaucratic systems or large institutions with centralized control (such as hospitals). Consistent with results, we also discuss the key benefits and possible risks of adopting blockchain technology in HIE efforts. This research can serve as a foundation for future studies in the domain of blockchain-based HIE.

## Appendix

Multimedia Appendix 1 Experiments: scenarios and questions.

Multimedia Appendix 2 Respondent characteristics across the 16 experiments.

## Abbreviations

ANOVA: analysis of variance
e-commerce: electronic commerce
EHR: electronic health record
HIE: health information exchange
HIPAA: Health Insurance Portability and Accountability Act
IS: information systems
IT: information technology
TAM: technology acceptance model
UTAUT: unified theory of acceptance and use of technology
