# Supplementary material for: The Potential of Blockchain Technology for Health Information Exchange: Experimental Study From Patients’ Perspectives
Source: J Med Internet Res. 2019 Jun 20;21(6):e14184. doi: 10.2196/14184 (PMC6610459; doi:10.2196/14184)
Supplement: Multimedia Appendix 1 [file jmir_v21i6e14184_app1.docx]

**Multimedia Appendix 1:**

**Experiments: scenarios and questions**

**Experiment one:**

Dear participants,

Please read the provided case scenario first, then answer the following questions accordingly.

Thank you very much for your time.

Case Scenario 1:

You are entering your doctor’s room at hospital A with having one major health symptom. After discussing your symptoms, your doctor says “Our organization is participating in a health information exchange (HIE) initiative meaning we are sharing your electronic health information (as required) with other healthcare providers such as other hospitals, physician practices, and laboratories within or outside of our network (regional or national). The model we use for sharing your information is the direct exchange model that automates point-to-point processes in which a provider can send patient data to a known recipient (another provider) using a secure network. Thus, this model facilitates the process of provider-to-provider electronic exchange of patient medical records. This will prevent you from carrying your health information later and make health care process quite efficient. “Are you willing to opt-in to the exchange method in this consent form?”

Type of health information that we may share:

Records relating to genetic information, mental health information, sexual health diseases, substance abuse, and addiction will be shared.

Privacy policy:

Then, the provider asks you to read the privacy policy related to information exchange practices.

· The privacy policy clearly articulates what health information is collected and exchanged, what the purpose of data exchange is, how such information will be used internally, and whether patient data will be disclosed to third parties.

· The privacy policy clearly indicates that patients will be given the options about how the collected personal information would be used. It delineates the consent process and permission requirements.

· The privacy policy clearly defines the patients’ rights to view their own personal data and check whether such data is accurate and complete.

· The privacy policy clearly defines the required steps and actions that should be taken by organizations to ensure security and integrity of consumers’ personal information.

· It clarifies the acceptable duration of keeping and processing shared health information for healthcare providers.

· It also articulates the reasonable steps to permanently delete shared personal data if it is no longer required for the consented purpose.

· The privacy policy articulates which national/international mechanisms, guidelines, and instruments are in place to enforce principles of privacy protection.

· This privacy policy statement clearly explains the provider’s policy concerning information that is provided by patients and makes an explicit guarantee that the information will be protected from unauthorized access or unanticipated secondary use by other organization, company, or individual.

**Experiment two:**

Dear participants,

Please read the provided case scenario first, then answer the following questions accordingly.

Thank you very much for your time.

Case scenario 2:

You are entering your doctor’s room at hospital B with having one major health symptom. After discussing your symptoms, your doctor says “Our organization is participating in a health information exchange (HIE) network meaning we are sharing your electronic health information (as required) with other healthcare providers such as other hospitals, physician practices, and laboratories, within or outside of our network (regional or national). The model we use for sharing your information is the non-direct model in which patient data are aggregated from multiple healthcare institutions in a central repository. Then, providers can send a request to the central repository in order to access patient medical records. By doing that, physicians can release patients’ information to a hub where other physicians can get access to it through a look-up process. This will prevent you from carrying your health information later and make health care process quite efficient. “Are you willing to opt-in to this exchange method in this consent form?”

Type of health information that we may share:

Records relating to genetic information, mental health information, sexual health diseases, substance abuse, and addiction will be shared.

Privacy policy:

Then, the provider asks you to read the privacy policy related to information exchange practices.

· The privacy policy clearly articulates what health information is collected and exchanged, what the purpose of data exchange is, how such information will be used internally, and whether patient data will be disclosed to third parties.

· The privacy policy clearly indicates that patients will be given the options about how the collected personal information would be used. It delineates the consent process and permission requirements.

· The privacy policy clearly defines the patients’ rights to view their own personal data and check whether such data is accurate and complete.

· The privacy policy clearly defines the required steps and actions that should be taken by organizations to ensure security and integrity of consumers’ personal information.

· It clarifies the acceptable duration of keeping and processing shared health information for healthcare providers.

· It also articulates the reasonable steps to permanently delete shared personal data if it is no longer required for the consented purpose.

· The privacy policy articulates which national/international mechanisms, guidelines, and instruments are in place to enforce principles of privacy protection.

· This privacy policy statement clearly explains the provider’s policy concerning information that is provided by patients and makes an explicit guarantee that the information will be protected from unauthorized access or unanticipated secondary use by other organization, company, or individual.

**Experiment three:**

Dear participants,

Please read the provided case scenario first, then answer the following questions accordingly.

Thank you very much for your time.

Case scenario 3:

You are entering your doctor’s room at hospital C with having one major health symptom. After discussing your symptoms, your doctor says “Our organization is participating in a health information exchange (HIE) project meaning we are sharing your electronic health information (as required) with other healthcare providers such as other hospitals, physician practices, and laboratories, within or outside of our network (regional or national). In this model your health information is saved in the hospital database. The model we use for sharing your information is the patient-centered exchange in which patient data and laboratory results, which are stored in the hospital's database, are delivered to the patient. Then, the patient can share it with any providers as required. This will prevent you from carrying your health information later and make health care process quite efficient. Are you willing to opt-in to this exchange method in this consent form?”

Type of health information that we may share:

Records relating to genetic information, mental health information, sexual health diseases, substance abuse, and addiction will be shared.

Privacy policy:

Then, the provider asks you to read the privacy policy related to information exchange practices.

· The privacy policy clearly articulates what health information is collected and exchanged, what the purpose of data exchange is, how such information will be used internally, and whether patient data will be disclosed to third parties.

· The privacy policy clearly indicates that patients will be given the options about how the collected personal information would be used. It delineates the consent process and permission requirements.

· The privacy policy clearly defines the patients’ rights to view their own personal data and check whether such data is accurate and complete.

· The privacy policy clearly defines the required steps and actions that should be taken by organizations to ensure security and integrity of consumers’ personal information.

· It clarifies the acceptable duration of keeping and processing shared health information for healthcare providers.

· It also articulates the reasonable steps to permanently delete shared personal data if it is no longer required for the consented purpose.

· The privacy policy articulates which national/international mechanisms, guidelines, and instruments are in place to enforce principles of privacy protection.

· This privacy policy statement clearly explains the provider’s policy concerning information that is provided by patients and makes an explicit guarantee that the information will be protected from unauthorized access or unanticipated secondary use by other organization, company, or individual.

**Experiment four:**

Dear participants,

Please read the provided case scenario first, then answer the following questions accordingly.

Thank you very much for your time.

Case scenario 4:

You are entering your doctor’s room at hospital D with having one major health symptom. After discussing your symptoms, your doctor says "A decentralized model of sharing health data is available now to patients in the US. Using this model, patients own and control their own health data. They can share all or part of their health information with other providers “. He continues: "As this model is based on a peer to peer network, you will be the owner of your data and can share all or part of it in a network which is not controlled or monitored by any hospitals or healthcare organizations. This model remove the need of middlemen or central authorities in the sharing process. Patients can provide permissions to other providers in order to check and view their health data. Sharing health data with other organizations will be possible without any interactions/influences from a specific hospital or provider. This method makes the data transaction more secure and makes it immune to hackers. Are you willing to opt-in to this exchange method in this consent form?”

Type of health information that we may share:

Records relating to genetic information, mental health information, sexual health diseases, substance abuse, and addiction will be shared.

Privacy policy:

Then, the provider asks you to read the privacy policy related to information exchange practices.

· The privacy policy clearly articulates what health information is collected and exchanged, what the purpose of data exchange is, how such information will be used internally, and whether patient data will be disclosed to third parties.

· The privacy policy clearly indicates that patients will be given the options about how the collected personal information would be used. It delineates the consent process and permission requirements.

· The privacy policy clearly defines the patients’ rights to view their own personal data and check whether such data is accurate and complete.

· The privacy policy clearly defines the required steps and actions that should be taken by organizations to ensure security and integrity of consumers’ personal information.

· It clarifies the acceptable duration of keeping and processing shared health information for healthcare providers.

· It also articulates the reasonable steps to permanently delete shared personal data if it is no longer required for the consented purpose.

· The privacy policy articulates which national/international mechanisms, guidelines, and instruments are in place to enforce principles of privacy protection.

· This privacy policy statement clearly explains the provider’s policy concerning information that is provided by patients and makes an explicit guarantee that the information will be protected from unauthorized access or unanticipated secondary use by other organization, company, or individual.

**Experiment five:**

Dear participants,

Please read the provided case scenario first, then answer the following questions accordingly.

Thank you very much for your time.

Case scenario 5:

You are entering your doctor’s room at hospital A with having one major health symptom. After discussing your symptoms, your doctor says “Our organization is participating in a health information exchange (HIE) initiative meaning we are sharing your electronic health information (as required) with other healthcare providers such as other hospitals, physician practices, and laboratories within or outside of our network (regional or national). The model we use for sharing your information is the direct exchange model that automates point-to-point processes in which a provider can send patient data to a known recipient (another provider) using a secure network. Thus, this model facilitates the process of provider-to-provider electronic exchange of patient medical records. This will prevent you from carrying your health information later and make health care process quite efficient. “Are you willing to opt-in to the exchange method in this consent form?”

Type of health information that we may share:

Information about your current health statues (e.g.: Fitness, diet, diseases and treatments) or past medical/health information (e.g.: list of vaccinations, medications used, etc.) will be shared.

Privacy policy:

Then, the provider asks you to read the privacy policy related to information exchange practices.

· The privacy policy clearly articulates what health information is collected and exchanged, what the purpose of data exchange is, how such information will be used internally, and whether patient data will be disclosed to third parties.

· The privacy policy clearly indicates that patients will be given the options about how the collected personal information would be used. It delineates the consent process and permission requirements.

· The privacy policy clearly defines the patients’ rights to view their own personal data and check whether such data is accurate and complete.

· The privacy policy clearly defines the required steps and actions that should be taken by organizations to ensure security and integrity of consumers’ personal information.

· It clarifies the acceptable duration of keeping and processing shared health information for healthcare providers.

· It also articulates the reasonable steps to permanently delete shared personal data if it is no longer required for the consented purpose.

· The privacy policy articulates which national/international mechanisms, guidelines, and instruments are in place to enforce principles of privacy protection.

· This privacy policy statement clearly explains the provider’s policy concerning information that is provided by patients and makes an explicit guarantee that the information will be protected from unauthorized access or unanticipated secondary use by other organization, company, or individual.

**Experiment six:**

Dear participants,

Please read the provided case scenario first, then answer the following questions accordingly.

Thank you very much for your time.

Case scenario 6:

You are entering your doctor’s room at hospital B with having one major health symptom. After discussing your symptoms, your doctor says “Our organization is participating in a health information exchange (HIE) network meaning we are sharing your electronic health information (as required) with other healthcare providers such as other hospitals, physician practices, and laboratories, within or outside of our network (regional or national). The model we use for sharing your information is the non-direct model in which patient data are aggregated from multiple healthcare institutions in a central repository. Then, providers can send a request to the central repository in order to access patient medical records. By doing that, physicians can release patients’ information to a hub where other physicians can get access to it through a lookup process. This will prevent you from carrying your health information later and make health care process quite efficient. “Are you willing to opt-in to this exchange method in this consent form?”

Type of health information that we may share:

Information about your current health statues (e.g.: Fitness, diet, diseases and treatments) or past medical/health information (e.g.: list of vaccinations, medications used, etc.) will be shared.

Privacy policy:

Then, the provider asks you to read the privacy policy related to information exchange practices.

· The privacy policy clearly articulates what health information is collected and exchanged, what the purpose of data exchange is, how such information will be used internally, and whether patient data will be disclosed to third parties.

· The privacy policy clearly indicates that patients will be given the options about how the collected personal information would be used. It delineates the consent process and permission requirements.

· The privacy policy clearly defines the patients’ rights to view their own personal data and check whether such data is accurate and complete.

· The privacy policy clearly defines the required steps and actions that should be taken by organizations to ensure security and integrity of consumers’ personal information.

· It clarifies the acceptable duration of keeping and processing shared health information for healthcare providers.

· It also articulates the reasonable steps to permanently delete shared personal data if it is no longer required for the consented purpose.

· The privacy policy articulates which national/international mechanisms, guidelines, and instruments are in place to enforce principles of privacy protection.

· This privacy policy statement clearly explains the provider’s policy concerning information that is provided by patients and makes an explicit guarantee that the information will be protected from unauthorized access or unanticipated secondary use by other organization, company, or individual.

**Experiment seven:**

Dear participants,

Please read the provided case scenario first, then answer the following questions accordingly.

Thank you very much for your time.

Case scenario 7:

You are entering your doctor’s room at hospital C with having one major health symptom. After discussing your symptoms, your doctor says “Our organization is participating in a health information exchange (HIE) project meaning we are sharing your electronic health information (as required) with other healthcare providers such as other hospitals, physician practices, and laboratories, within or outside of our network (regional or national). In this model your health information is saved in the hospital database. The model we use for sharing your information is the patient-centered exchange in which patient data and laboratory results, which are stored in the hospital's database, are delivered to the patient. Then, the patient can share it with any providers as required. This will prevent you from carrying your health information later and make health care process quite efficient. Are you willing to opt-in to this exchange method in this consent form?”

Type of health information that we may share:

Information about your current health statues (e.g.: Fitness, diet, diseases and treatments) or past medical/health information (e.g.: list of vaccinations, medications used, etc.) will be shared.

Privacy policy:

Then, the provider asks you to read the privacy policy related to information exchange practices.

· The privacy policy clearly articulates what health information is collected and exchanged, what the purpose of data exchange is, how such information will be used internally, and whether patient data will be disclosed to third parties.

· The privacy policy clearly indicates that patients will be given the options about how the collected personal information would be used. It delineates the consent process and permission requirements.

· The privacy policy clearly defines the patients’ rights to view their own personal data and check whether such data is accurate and complete.

· The privacy policy clearly defines the required steps and actions that should be taken by organizations to ensure security and integrity of consumers’ personal information.

· It clarifies the acceptable duration of keeping and processing shared health information for healthcare providers.

· It also articulates the reasonable steps to permanently delete shared personal data if it is no longer required for the consented purpose.

· The privacy policy articulates which national/international mechanisms, guidelines, and instruments are in place to enforce principles of privacy protection.

· This privacy policy statement clearly explains the provider’s policy concerning information that is provided by patients and makes an explicit guarantee that the information will be protected from unauthorized access or unanticipated secondary use by other organization, company, or individual.

**Experiment eight:**

Dear participants,

Please read the provided case scenario first, then answer the following questions accordingly.

Thank you very much for your time.

Case scenario 8:

You are entering your doctor’s room at hospital D with having one major health symptom. After discussing your symptoms, your doctor says "A decentralized model of sharing health data is available now to patients in the US. Using this model, patients own and control their own health data. They can share all or part of their health information with other providers “. He continues: "As this model is based on a peer to peer network, you will be the owner of your data and can share all or part of it in a network which is not controlled or monitored by any hospitals or healthcare organizations. This model remove the need of middlemen or central authorities in the sharing process. Patients can provide permissions to other providers in order to check and view their health data. Sharing health data with other organizations will be possible without any interactions/influences from a specific hospital or provider. This method makes the data transaction more secure and makes it immune to hackers. Are you willing to opt-in to this exchange method in this consent form?”

Type of health information that we share:

Information about your current health statues (e.g.: Fitness, diet, diseases and treatments) or past medical/health information (e.g.: list of vaccinations, medications used, etc.) will be shared.

Privacy policy:

Then, the provider asks you to read the privacy policy related to information exchange practices.

· The privacy policy clearly articulates what health information is collected and exchanged, what the purpose of data exchange is, how such information will be used internally, and whether patient data will be disclosed to third parties.

· The privacy policy clearly indicates that patients will be given the options about how the collected personal information would be used. It delineates the consent process and permission requirements.

· The privacy policy clearly defines the patients’ rights to view their own personal data and check whether such data is accurate and complete.

· The privacy policy clearly defines the required steps and actions that should be taken by organizations to ensure security and integrity of consumers’ personal information.

· It clarifies the acceptable duration of keeping and processing shared health information for healthcare providers.

· It also articulates the reasonable steps to permanently delete shared personal data if it is no longer required for the consented purpose.

· The privacy policy articulates which national/international mechanisms, guidelines, and instruments are in place to enforce principles of privacy protection.

· This privacy policy statement clearly explains the provider’s policy concerning information that is provided by patients and makes an explicit guarantee that the information will be protected from unauthorized access or unanticipated secondary use by other organization, company, or individual.

**Experiment nine:**

Dear participants,

Please read the provided case scenario first, then answer the following questions accordingly.

Thank you very much for your time.

Case scenario 9:

You are entering your doctor’s room at hospital A with having one major health symptom. After discussing your symptoms, your doctor says “Our organization is participating in a health information exchange (HIE) initiative meaning we are sharing your electronic health information (as required) with other healthcare providers such as other hospitals, physician practices, and laboratories within or outside of our network (regional or national). The model we use for sharing your information is the direct exchange model that automates point-to-point processes in which a provider can send patient data to a known recipient (another provider) using a secure network. Thus, this model facilitates the process of provider-to-provider electronic exchange of patient medical records. This will prevent you from carrying your health information later and make health care process quite efficient. “Are you willing to opt-in to the exchange method in this consent form?”

Type of health information that we may share:

Records relating to genetic information, mental health information, sexual health diseases, substance abuse, and addiction will be shared.

Privacy policy:

Then, the provider asks you to read the privacy policy related to information exchange practices.

· However, the privacy policy does not articulate what health information is collected and exchanged, what the purpose of data exchange is, how such information will be used internally, and whether patient data will be disclosed to third parties.

· The privacy policy does not indicate that patients will be given the options about how the collected personal information would be used.

· It does not delineate the consent process and permission requirements.

· The privacy policy does not define the patients’ rights to view their own personal data and check whether such data is accurate and complete.

· The privacy policy does not define the required steps and actions that should be taken by organizations to ensure security and integrity of consumers’ personal information.

· It does not clarify the acceptable duration of keeping and processing shared health information for healthcare providers. It also does not articulate the reasonable steps to permanently delete shared personal data if it is no longer required for the consented purpose.

· It is also not clear which national/international mechanisms, guidelines, and instruments are in place to enforce principles of privacy protection.

· This privacy policy statement explains the provider’s policy concerning information that is provided by the patients, but does not offer any guarantee with respect to protecting personal information.

**Experiment ten:**

Dear participants,

Please read the provided case scenario first, then answer the following questions accordingly.

Thank you very much for your time.

Case scenario 10:

You are entering your doctor’s room at hospital B with having one major health symptom. After discussing your symptoms, your doctor says “Our organization is participating in a health information exchange (HIE) network meaning we are sharing your electronic health information (as required) with other healthcare providers such as other hospitals, physician practices, and laboratories, within or outside of our network (regional or national). The model we use for sharing your information is the non-direct model in which patient data are aggregated from multiple healthcare institutions in a central repository. Then, providers can send a request to the central repository in order to access patient medical records. By doing that, physicians can release patients’ information to a hub where other physicians can get access to it through a lookup process. This will prevent you from carrying your health information later and make health care process quite efficient. “Are you willing to opt-in to this exchange method in this consent form?”

Type of health information that we may share:

Records relating to genetic information, mental health information, sexual health diseases, substance abuse, and addiction will be shared.

Privacy policy:

Then, the provider asks you to read the privacy policy related to information exchange practices.

· However, the privacy policy does not articulate what health information is collected and exchanged, what the purpose of data exchange is, how such information will be used internally, and whether patient data will be disclosed to third parties.

· The privacy policy does not indicate that patients will be given the options about how the collected personal information would be used.

· It does not delineate the consent process and permission requirements.

· The privacy policy does not define the patients’ rights to view their own personal data and check whether such data is accurate and complete.

· The privacy policy does not define the required steps and actions that should be taken by organizations to ensure security and integrity of consumers’ personal information.

· It does not clarify the acceptable duration of keeping and processing shared health information for healthcare providers. It also does not articulate the reasonable steps to permanently delete shared personal data if it is no longer required for the consented purpose.

· It is also not clear which national/international mechanisms, guidelines, and instruments are in place to enforce principles of privacy protection.

· This privacy policy statement explains the provider’s policy concerning information that is provided by the patients, but does not offer any guarantee with respect to protecting personal information.

**Experiment eleven:**

Dear participants,

Please read the provided case scenario first, then answer the following questions accordingly.

Thank you very much for your time.

Case scenario 11:

You are entering your doctor’s room at hospital C with having one major health symptom. After discussing your symptoms, your doctor says “Our organization is participating in a health information exchange (HIE) project meaning we are sharing your electronic health information (as required) with other healthcare providers such as other hospitals, physician practices, and laboratories, within or outside of our network (regional or national). In this model your health information is saved in the hospital database. The model we use for sharing your information is the patient-centered exchange in which patient data and laboratory results, which are stored in the hospital's database, are delivered to the patient. Then, the patient can share it with any providers as required. This will prevent you from carrying your health information later and make health care process quite efficient. Are you willing to opt-in to this exchange method in this consent form?”

Type of health information that we may share:

Records relating to genetic information, mental health information, sexual health diseases, substance abuse, and addiction will be shared.

Privacy policy:

Then, the provider asks you to read the privacy policy related to information exchange practices.

· However, the privacy policy does not articulate what health information is collected and exchanged, what the purpose of data exchange is, how such information will be used internally, and whether patient data will be disclosed to third parties.

· The privacy policy does not indicate that patients will be given the options about how the collected personal information would be used.

· It does not delineate the consent process and permission requirements.

· The privacy policy does not define the patients’ rights to view their own personal data and check whether such data is accurate and complete.

· The privacy policy does not define the required steps and actions that should be taken by organizations to ensure security and integrity of consumers’ personal information.

· It does not clarify the acceptable duration of keeping and processing shared health information for healthcare providers. It also does not articulate the reasonable steps to permanently delete shared personal data if it is no longer required for the consented purpose.

· It is also not clear which national/international mechanisms, guidelines, and instruments are in place to enforce principles of privacy protection.

· This privacy policy statement explains the provider’s policy concerning information that is provided by the patients, but does not offer any guarantee with respect to protecting personal information.

**Experiment twelve:**

Dear participants,

Please read the provided case scenario first, then answer the following questions accordingly.

Thank you very much for your time.

Case scenario 12:

You are entering your doctor’s room at hospital D with having one major health symptom. After discussing your symptoms, your doctor says "A decentralized model of sharing health data is available now to patients in the US. Using this model, patients own and control their own health data. They can share all or part of their health information with other providers “. He continues: "As this model is based on a peer to peer network, you will be the owner of your data and can share all or part of it in a network which is not controlled or monitored by any hospitals or healthcare organizations. This model remove the need of middlemen or central authorities in the sharing process. Patients can provide permissions to other providers in order to check and view their health data. Sharing health data with other organizations will be possible without any interactions/influences from a specific hospital or provider. This method makes the data transaction more secure and makes it immune to hackers. Are you willing to opt-in to this exchange method in this consent form?”

Type of health information that we may share:

Records relating to genetic information, mental health information, sexual health diseases, substance abuse, and addiction will be shared.

Privacy policy

Then, the provider asks you to read the privacy policy related to information exchange practices.

· However, the privacy policy does not articulate what health information is collected and exchanged, what the purpose of data exchange is, how such information will be used internally, and whether patient data will be disclosed to third parties.

· The privacy policy does not indicate that patients will be given the options about how the collected personal information would be used.

· It does not delineate the consent process and permission requirements.

· The privacy policy does not define the patients’ rights to view their own personal data and check whether such data is accurate and complete.

· The privacy policy does not define the required steps and actions that should be taken by organizations to ensure security and integrity of consumers’ personal information.

· It does not clarify the acceptable duration of keeping and processing shared health information for healthcare providers. It also does not articulate the reasonable steps to permanently delete shared personal data if it is no longer required for the consented purpose.

· It is also not clear which national/international mechanisms, guidelines, and instruments are in place to enforce principles of privacy protection.

· This privacy policy statement explains the provider’s policy concerning information that is provided by the patients, but does not offer any guarantee with respect to protecting personal information.

**Experiment thirteen:**

Dear participants,

Please read the provided case scenario first, then answer the following questions accordingly.

Thank you very much for your time.

Case scenario 13:

You are entering your doctor’s room at hospital A with having one major health symptom. After discussing your symptoms, your doctor says “Our organization is participating in a health information exchange (HIE) initiative meaning we are sharing your electronic health information (as required) with other healthcare providers such as other hospitals, physician practices, and laboratories within or outside of our network (regional or national). The model we use for sharing your information is the direct exchange model that automates point-to-point processes in which a provider can send patient data to a known recipient (another provider) using a secure network. Thus, this model facilitates the process of provider-to-provider electronic exchange of patient medical records. This will prevent you from carrying your health information later and make health care process quite efficient. “Are you willing to opt-in to the exchange method in this consent form?”

Type of health information that we may share:

Information about your current health statues (e.g.: Fitness, diet, diseases and treatments) or past medical/health information (e.g.: list of vaccinations, medications used, etc.) will be shared.

Privacy policy:

Then, the provider asks you to read the privacy policy related to information exchange practices.

· However, the privacy policy does not articulate what health information is collected and exchanged, what the purpose of data exchange is, how such information will be used internally, and whether patient data will be disclosed to third parties.

· The privacy policy does not indicate that patients will be given the options about how the collected personal information would be used.

· It does not delineate the consent process and permission requirements.

· The privacy policy does not define the patients’ rights to view their own personal data and check whether such data is accurate and complete.

· The privacy policy does not define the required steps and actions that should be taken by organizations to ensure security and integrity of consumers’ personal information.

· It does not clarify the acceptable duration of keeping and processing shared health information for healthcare providers. It also does not articulate the reasonable steps to permanently delete shared personal data if it is no longer required for the consented purpose.

· It is also not clear which national/international mechanisms, guidelines, and instruments are in place to enforce principles of privacy protection.

· This privacy policy statement explains the provider’s policy concerning information that is provided by the patients, but does not offer any guarantee with respect to protecting personal information.

**Experiment fourteen:**

Dear participants,

Please read the provided case scenario first, then answer the following questions accordingly.

Thank you very much for your time.

Case scenario 14:

You are entering your doctor’s room at hospital B with having one major health symptom. After discussing your symptoms, your doctor says “Our organization is participating in a health information exchange (HIE) network meaning we are sharing your electronic health information (as required) with other healthcare providers such as other hospitals, physician practices, and laboratories, within or outside of our network (regional or national). The model we use for sharing your information is the non-direct model in which patient data are aggregated from multiple healthcare institutions in a central repository. Then, providers can send a request to the central repository in order to access patient medical records. By doing that, physicians can release patients’ information to a hub where other physicians can get access to it through a lookup process. This will prevent you from carrying your health information later and make health care process quite efficient. “Are you willing to opt-in to this exchange method in this consent form?”

Type of health information that we may share:

Information about your current health statues (e.g.: Fitness, diet, diseases and treatments) or past medical/health information (e.g.: list of vaccinations, medications used, etc.) will be shared.

Privacy policy:

Then, the provider asks you to read the privacy policy related to information exchange practices.

· However, the privacy policy does not articulate what health information is collected and exchanged, what the purpose of data exchange is, how such information will be used internally, and whether patient data will be disclosed to third parties.

· The privacy policy does not indicate that patients will be given the options about how the collected personal information would be used.

· It does not delineate the consent process and permission requirements.

· The privacy policy does not define the patients’ rights to view their own personal data and check whether such data is accurate and complete.

· The privacy policy does not define the required steps and actions that should be taken by organizations to ensure security and integrity of consumers’ personal information.

· It does not clarify the acceptable duration of keeping and processing shared health information for healthcare providers. It also does not articulate the reasonable steps to permanently delete shared personal data if it is no longer required for the consented purpose.

· It is also not clear which national/international mechanisms, guidelines, and instruments are in place to enforce principles of privacy protection.

· This privacy policy statement explains the provider’s policy concerning information that is provided by the patients, but does not offer any guarantee with respect to protecting personal information.

**Experiment fifteen:**

Dear participants,

Please read the provided case scenario first, then answer the following questions accordingly.

Thank you very much for your time.

Case scenario 15:

You are entering your doctor’s room at hospital C with having one major health symptom. After discussing your symptoms, your doctor says “Our organization is participating in a health information exchange (HIE) project meaning we are sharing your electronic health information (as required) with other healthcare providers such as other hospitals, physician practices, and laboratories, within or outside of our network (regional or national). In this model your health information is saved in the hospital database. The model we use for sharing your information is the patient-centered exchange in which patient data and laboratory results, which are stored in the hospital's database, are delivered to the patient. Then, the patient can share it with any providers as required. This will prevent you from carrying your health information later and make health care process quite efficient. Are you willing to opt-in to this exchange method in this consent form?”

Type of health information that we may share:

Information about your current health statues (e.g.: Fitness, diet, diseases and treatments) or past medical/health information (e.g.: list of vaccinations, medications used, etc.) will be shared.

Privacy policy:

Then, the provider asks you to read the privacy policy related to information exchange practices.

· However, the privacy policy does not articulate what health information is collected and exchanged, what the purpose of data exchange is, how such information will be used internally, and whether patient data will be disclosed to third parties.

· The privacy policy does not indicate that patients will be given the options about how the collected personal information would be used.

· It does not delineate the consent process and permission requirements.

· The privacy policy does not define the patients’ rights to view their own personal data and check whether such data is accurate and complete.

· The privacy policy does not define the required steps and actions that should be taken by organizations to ensure security and integrity of consumers’ personal information.

· It does not clarify the acceptable duration of keeping and processing shared health information for healthcare providers. It also does not articulate the reasonable steps to permanently delete shared personal data if it is no longer required for the consented purpose.

· It is also not clear which national/international mechanisms, guidelines, and instruments are in place to enforce principles of privacy protection.

· This privacy policy statement explains the provider’s policy concerning information that is provided by the patients, but does not offer any guarantee with respect to protecting personal information.

**Experiment sixteen:**

Dear participants,

Please read the provided case scenario first, then answer the following questions accordingly.

Thank you very much for your time.

Case scenario 16:

You are entering your doctor’s room at hospital D with having one major health symptom. After discussing your symptoms, your doctor says "A decentralized model of sharing health data is available now to patients in the US. Using this model, patients own and control their own health data. They can share all or part of their health information with other providers “. He continues: "As this model is based on a peer to peer network, you will be the owner of your data and can share all or part of it in a network which is not controlled or monitored by any hospitals or healthcare organizations. This model remove the need of middlemen or central authorities in the sharing process. Patients can provide permissions to other providers in order to check and view their health data. Sharing health data with other organizations will be possible without any interactions/influences from a specific hospital or provider. This method makes the data transaction more secure and makes it immune to hackers. Are you willing to opt-in to this exchange method in this consent form?”

Type of health information that we may share:

Information about your current health statues (e.g.: Fitness, diet, diseases and treatments) or past medical/health information (e.g.: list of vaccinations, medications used, etc.) will be shared.

Privacy policy:

Then, the provider asks you to read the privacy policy related to information exchange practices.

· However, the privacy policy does not articulate what health information is collected and exchanged, what the purpose of data exchange is, how such information will be used internally, and whether patient data will be disclosed to third parties.

· The privacy policy does not indicate that patients will be given the options about how the collected personal information would be used.

· It does not delineate the consent process and permission requirements.

· The privacy policy does not define the patients’ rights to view their own personal data and check whether such data is accurate and complete.

· The privacy policy does not define the required steps and actions that should be taken by organizations to ensure security and integrity of consumers’ personal information.

· It does not clarify the acceptable duration of keeping and processing shared health information for healthcare providers. It also does not articulate the reasonable steps to permanently delete shared personal data if it is no longer required for the consented purpose.

· It is also not clear which national/international mechanisms, guidelines, and instruments are in place to enforce principles of privacy protection.

· This privacy policy statement explains the provider’s policy concerning information that is provided by the patients, but does not offer any guarantee with respect to protecting personal information.

**Shared questions across the sixteen experimental conditions:**

Please answer the following questions considering the case scenario, type of health information, and the transparency of the privacy policy:

Q1. I am concerned that...

-Using (*the mentioned exchange mechanism*) helps health care entities collect too much personal information from me.

Strongly agree Somewhat agree Neither agree nor disagree Somewhat disagree Strongly disagree

-If (*the mentioned exchange mechanism*) is used, it is likely that health care entities can use my health information for other purposes without my knowledge and authorization.

Strongly agree Somewhat agree Neither agree nor disagree Somewhat disagree Strongly disagree

-If (*the mentioned exchange mechanism*) is used, it is likely that my health information will be shared with other entities without my authorization.

Strongly agree Somewhat agree Neither agree nor disagree Somewhat disagree Strongly disagree

-If (*the mentioned exchange mechanism*) is used, it is likely that unauthorized people have access to my health information.

Strongly agree Somewhat agree Neither agree nor disagree Somewhat disagree Strongly disagree

-If (*the mentioned exchange mechanism*) is used, I am concerned about the privacy of my health information during an exchange transaction.

Strongly agree Somewhat agree Neither agree nor disagree Somewhat disagree Strongly disagree

-If (*the mentioned exchange mechanism*) is used, my health information can be sold easier to others without my permission.

Strongly agree Somewhat agree Neither agree nor disagree Somewhat disagree Strongly disagree

-If (*the mentioned exchange mechanism*) is used, it is likely that the health information exchanged to health care entities is inaccurate.

Strongly agree Somewhat agree Neither agree nor disagree Somewhat disagree Strongly disagree

Q2.

- I believe that the mentioned exchange mechanism is a real expert system in information sharing

Strongly agree Somewhat agree Neither agree nor disagree Somewhat disagree Strongly disagree

- I believe that the mentioned exchange mechanism is capable and competent in sharing health information electronically

Strongly agree Somewhat agree Neither agree nor disagree Somewhat disagree Strongly disagree

- I believe that the mentioned exchange mechanism is able to adapt to specific and unforeseen situations

Strongly agree Somewhat agree Neither agree nor disagree Somewhat disagree Strongly disagree

- I believe that the mentioned exchange mechanism has a standard of competency to carry out information sharing

Strongly agree Somewhat agree Neither agree nor disagree Somewhat disagree Strongly disagree

Q3.

- I think the mentioned exchange mechanism is honest

Strongly agree Somewhat agree Neither agree nor disagree Somewhat disagree Strongly disagree

- I consider the mentioned exchange mechanism to be of integrity

Strongly agree Somewhat agree Neither agree nor disagree Somewhat disagree Strongly disagree

- I believe that the promises made by the mentioned exchange mechanism are likely to be reliable

Strongly agree Somewhat agree Neither agree nor disagree Somewhat disagree Strongly disagree

- I expect that the mentioned exchange mechanism keeps promises it makes

Strongly agree Somewhat agree Neither agree nor disagree Somewhat disagree Strongly disagree

- The mentioned exchange mechanism does not make false claims and information

Strongly agree Somewhat agree Neither agree nor disagree Somewhat disagree Strongly disagree

Q4. Please answer the following questions about your opt-in intention to the given scenario:

-I accept to opt-in toward (*the mentioned exchange mechanism*) to exchange my health information among health care entities

Strongly agree Somewhat agree Neither agree nor disagree Somewhat disagree Strongly disagree

-Using (*the mentioned exchange mechanism*) is something I would support

Strongly agree Somewhat agree Neither agree nor disagree Somewhat disagree Strongly disagree

-I would like my health care providers to adopt (*the mentioned exchange mechanism*) to share my health information

Strongly agree Somewhat agree Neither agree nor disagree Somewhat disagree Strongly disagree

-I will endorse my physicians to use (*the mentioned exchange mechanism*) in their practice

Strongly agree Somewhat agree Neither agree nor disagree Somewhat disagree Strongly disagree

Q5. Please answer the following questions about your willingness to share your health information based on the given scenario:

- If (*the mentioned exchange mechanism*) is implemented, I am very likely to provide my health information in the future

Strongly agree Somewhat agree Neither agree nor disagree Somewhat disagree Strongly disagree

- Given that my health information may be shared in future with other providers through (*the mentioned exchange mechanism*), I am willing to disclose my health information

Strongly agree Somewhat agree Neither agree nor disagree Somewhat disagree Strongly disagree

- In the future, I am willing to provide my personal information to a physician who use (*the mentioned exchange mechanism*)

Strongly agree Somewhat agree Neither agree nor disagree Somewhat disagree Strongly disagree

- It is probable that I will release my health information to be exchanged through (*the mentioned exchange mechanism*)

Strongly agree Somewhat agree Neither agree nor disagree Somewhat disagree Strongly disagree

Q6. Please answer the following questions considering potential benefits of the mentioned exchange mechanism:

- I think the mentioned exchange mechanism can reduce additional efforts to carry my medical information

Strongly agree Somewhat agree Neither agree nor disagree Somewhat disagree Strongly disagree

- The mentioned exchange mechanism can save me money by not traveling across different care providers and copying medical records

Strongly agree Somewhat agree Neither agree nor disagree Somewhat disagree Strongly disagree

- I believe that the mentioned exchange mechanism handles information sharing very quickly

Strongly agree Somewhat agree Neither agree nor disagree Somewhat disagree Strongly disagree

- I think the mentioned exchange mechanism is very convenient

Strongly agree Somewhat agree Neither agree nor disagree Somewhat disagree Strongly disagree

- I think the mentioned exchange mechanism improves coordination and communication with doctors involved in my care

Strongly agree Somewhat agree Neither agree nor disagree Somewhat disagree Strongly disagree

- I think the mentioned exchange mechanism provides real time access to my health information

Strongly agree Somewhat agree Neither agree nor disagree Somewhat disagree Strongly disagree
