# Supplementary material for: The Potential of Blockchain Technology for Health Information Exchange: Experimental Study From Patients’ Perspectives
Source: J Med Internet Res. 2019 Jun 20;21(6):e14184. doi: 10.2196/14184 (PMC6610459; doi:10.2196/14184)
Supplement: Multimedia Appendix 2 [file jmir_v21i6e14184_app2.docx]

**Multimedia Appendix 2:**

**Respondent characteristics across the 16 experiments**

1. Gender:

|  | Male | | Female | | Total | |
| --- | --- | --- | --- | --- | --- | --- |
| Scenario | Count | % | Count | % | Count | % |
| 1 | 59 | 46.1% | 69 | 53.9% | 128 | 100.0% |
| 2 | 49 | 38.3% | 79 | 61.7% | 128 | 100.0% |
| 3 | 48 | 39.3% | 74 | 60.7% | 122 | 100.0% |
| 4 | 48 | 40.7% | 72 | 59.3% | 120 | 100.0% |
| 5 | 49 | 39.8% | 74 | 60.2% | 123 | 100.0% |
| 6 | 53 | 40.5% | 78 | 59.5% | 131 | 100.0% |
| 7 | 58 | 46.0% | 68 | 54.0% | 126 | 100.0% |
| 8 | 45 | 36.0% | 80 | 64.0% | 125 | 100.0% |
| 9 | 47 | 36.7% | 81 | 63.3% | 128 | 100.0% |
| 10 | 52 | 41.9% | 72 | 58.1% | 124 | 100.0% |
| 11 | 48 | 37.8% | 79 | 62.2% | 127 | 100.0% |
| 12 | 59 | 46.8% | 67 | 53.2% | 126 | 100.0% |
| 13 | 52 | 39.4% | 80 | 60.6% | 132 | 100.0% |
| 14 | 41 | 32.5% | 85 | 67.5% | 126 | 100.0% |
| 15 | 45 | 36.0% | 80 | 64.0% | 125 | 100.0% |
| 16 | 49 | 40.2% | 73 | 59.8% | 122 | 100.0% |
| Total | 802 | 39.9% | 1209 | 60.1% | 2013 | 100.0% |

|  | Value | df | p-value |
| --- | --- | --- | --- |
| Pearson Chi-Square | 12.187 | 15 | 0.66 |

2. Age:

|  | Under 20 | | 20 – 29 | | 30 - 39 | | 40 - 49 | | 50 - 59 | | 60 or older | | Total | |
| --- | --- | --- | --- | --- | --- | --- | --- | --- | --- | --- | --- | --- | --- | --- |
|  | Count | % | Count | % | Count | % | Count | % | Count | % | Count | % | Count |  |
| 1 | 0 | 0% | 35 | 27% | 51 | 40% | 18 | 14% | 16 | 13% | 8 | 6% | 128 | 100% |
| 2 | 1 | 1% | 41 | 32% | 49 | 38% | 21 | 16% | 10 | 8% | 6 | 5% | 128 | 100% |
| 3 | 0 | 0% | 31 | 25% | 44 | 36% | 26 | 21% | 13 | 11% | 8 | 7% | 122 | 100% |
| 4 | 1 | 1% | 34 | 29% | 42 | 36% | 20 | 16% | 16 | 14% | 7 | 5% | 120 | 100% |
| 5 | 5 | 4% | 33 | 27% | 45 | 37% | 19 | 15% | 19 | 15% | 2 | 2% | 123 | 100% |
| 6 | 4 | 3% | 41 | 31% | 39 | 30% | 30 | 23% | 7 | 5% | 10 | 8% | 131 | 100% |
| 7 | 2 | 2% | 39 | 31% | 40 | 32% | 20 | 16% | 14 | 11% | 11 | 9% | 126 | 100% |
| 8 | 0 | 0% | 45 | 36% | 49 | 39% | 9 | 7% | 12 | 10% | 10 | 8% | 125 | 100% |
| 9 | 2 | 2% | 34 | 27% | 50 | 39% | 16 | 13% | 17 | 13% | 9 | 7% | 128 | 100% |
| 10 | 2 | 2% | 40 | 32% | 36 | 29% | 26 | 21% | 17 | 14% | 3 | 2% | 124 | 100% |
| 11 | 0 | 0% | 31 | 24% | 50 | 39% | 21 | 17% | 12 | 9% | 13 | 10% | 127 | 100% |
| 12 | 1 | 1% | 42 | 33% | 42 | 33% | 23 | 18% | 11 | 9% | 7 | 6% | 126 | 100% |
| 13 | 0 | 0% | 42 | 32% | 40 | 30% | 20 | 15% | 21 | 16% | 9 | 7% | 132 | 100% |
| 14 | 0 | 0% | 34 | 27% | 46 | 37% | 25 | 20% | 14 | 11% | 7 | 6% | 126 | 100% |
| 15 | 3 | 2% | 31 | 25% | 57 | 46% | 20 | 16% | 8 | 6% | 6 | 5% | 125 | 100% |
| 16 | 0 | 0% | 35 | 29% | 45 | 37% | 22 | 18% | 13 | 11% | 7 | 6% | 122 | 100% |
| Total | 21 | 1% | 588 | 29% | 725 | 36% | 335 | 17% | 220 | 11% | 122 | 6% | 2013 | 100% |

|  | Value | df | p-value |
| --- | --- | --- | --- |
| Pearson Chi-Square | 92.871 | 75 | 0.08 |

3. Household Income:

|  | less than $25,000 | | $25,000-$49,999 | | $50,000-$74,999 | | $75,000-$99,999 | | $100,000 or more | | Total | |
| --- | --- | --- | --- | --- | --- | --- | --- | --- | --- | --- | --- | --- |
|  | Count | % | Count | % | Count | % | Count | % | Count | % | Count | % |
| 1 | 22 | 17% | 45 | 35% | 31 | 24% | 13 | 10% | 17 | 13% | 128 | 100% |
| 2 | 25 | 20% | 41 | 32% | 36 | 28% | 13 | 10% | 13 | 10% | 128 | 100% |
| 3 | 33 | 27% | 32 | 26% | 34 | 28% | 11 | 9% | 12 | 10% | 122 | 100% |
| 4 | 19 | 15% | 37 | 31% | 31 | 26% | 21 | 18% | 12 | 9% | 120 | 100% |
| 5 | 17 | 14% | 37 | 30% | 30 | 24% | 19 | 15% | 20 | 16% | 123 | 100% |
| 6 | 20 | 15% | 39 | 30% | 35 | 27% | 21 | 16% | 16 | 12% | 131 | 100% |
| 7 | 24 | 19% | 37 | 29% | 34 | 27% | 16 | 13% | 15 | 12% | 126 | 100% |
| 8 | 18 | 14% | 42 | 34% | 34 | 27% | 17 | 14% | 14 | 11% | 125 | 100% |
| 9 | 29 | 23% | 41 | 32% | 26 | 20% | 15 | 12% | 17 | 13% | 128 | 100% |
| 10 | 20 | 16% | 43 | 35% | 26 | 21% | 16 | 13% | 19 | 15% | 124 | 100% |
| 11 | 27 | 21% | 33 | 26% | 29 | 23% | 15 | 12% | 23 | 18% | 127 | 100% |
| 12 | 15 | 12% | 43 | 34% | 31 | 25% | 18 | 14% | 19 | 15% | 126 | 100% |
| 13 | 29 | 22% | 46 | 35% | 25 | 19% | 21 | 16% | 11 | 8% | 132 | 100% |
| 14 | 21 | 17% | 41 | 33% | 26 | 21% | 24 | 19% | 14 | 11% | 126 | 100% |
| 15 | 23 | 18% | 35 | 28% | 34 | 27% | 22 | 18% | 11 | 9% | 125 | 100% |
| 16 | 15 | 12% | 51 | 42% | 29 | 24% | 12 | 10% | 15 | 12% | 122 | 100% |
| Total | 356 | 18% | 643 | 32% | 491 | 24% | 274 | 14% | 247 | 12% | 2013 | 100% |

|  | Value | df | p-value |
| --- | --- | --- | --- |
| Pearson Chi-Square | 59.069 | 60 | 0.51 |

4. Level of Education

|  | Less than high school | | High school graduate | | Some college | | Associates degree | | Undergrad degree | | Doctorate | | Total | |
| --- | --- | --- | --- | --- | --- | --- | --- | --- | --- | --- | --- | --- | --- | --- |
|  | Count | % | Count | % | Count | % | Count | % | Count | % | Count | % | Count | % |
| 1 | 1 | 1% | 10 | 8% | 25 | 20% | 21 | 16% | 66 | 52% | 5 | 4% | 128 | 100% |
| 2 | 2 | 2% | 12 | 9% | 30 | 23% | 19 | 15% | 57 | 45% | 8 | 6% | 128 | 100% |
| 3 | 0 | 0% | 14 | 11% | 31 | 25% | 19 | 16% | 53 | 43% | 5 | 4% | 122 | 100% |
| 4 | 2 | 1% | 15 | 13% | 31 | 26% | 13 | 10% | 53 | 45% | 6 | 5% | 120 | 100% |
| 5 | 2 | 2% | 21 | 17% | 30 | 24% | 12 | 10% | 55 | 45% | 3 | 2% | 123 | 100% |
| 6 | 0 | 0% | 17 | 13% | 35 | 27% | 14 | 11% | 58 | 44% | 7 | 5% | 131 | 100% |
| 7 | 3 | 2% | 14 | 11% | 30 | 24% | 21 | 17% | 53 | 42% | 5 | 4% | 126 | 100% |
| 8 | 1 | 1% | 13 | 10% | 29 | 23% | 14 | 11% | 59 | 47% | 9 | 7% | 125 | 100% |
| 9 | 4 | 3% | 18 | 14% | 26 | 20% | 18 | 14% | 59 | 46% | 3 | 2% | 128 | 100% |
| 10 | 1 | 1% | 11 | 9% | 28 | 23% | 18 | 15% | 63 | 51% | 3 | 2% | 124 | 100% |
| 11 | 0 | 0% | 16 | 13% | 31 | 24% | 10 | 8% | 65 | 51% | 5 | 4% | 127 | 100% |
| 12 | 0 | 0% | 8 | 6% | 30 | 24% | 14 | 11% | 65 | 52% | 9 | 7% | 126 | 100% |
| 13 | 2 | 2% | 17 | 13% | 41 | 31% | 13 | 10% | 56 | 42% | 3 | 2% | 132 | 100% |
| 14 | 1 | 1% | 7 | 6% | 35 | 28% | 9 | 7% | 67 | 53% | 7 | 6% | 126 | 100% |
| 15 | 3 | 2% | 9 | 7% | 29 | 23% | 13 | 10% | 58 | 46% | 13 | 10% | 125 | 100% |
| 16 | 1 | 1% | 13 | 11% | 29 | 24% | 14 | 11% | 59 | 48% | 6 | 5% | 122 | 100% |
| Total | 22 | 1% | 215 | 11% | 490 | 24% | 241 | 12% | 946 | 47% | 97 | 5% | 2013 | 100% |

|  | Value | df | p-value |
| --- | --- | --- | --- |
| Pearson Chi-Square | 76.199 | 75 | 0.44 |

5. Employment Status:

|  | Employed full time | | Employed part time | | Unemployed | | Retired | | Student | | Total | |
| --- | --- | --- | --- | --- | --- | --- | --- | --- | --- | --- | --- | --- |
|  | Count | % | Count | % | Count | % | Count | % | Count | % | Count | % |
| 1 | 85 | 66% | 22 | 17% | 12 | 9% | 6 | 5% | 3 | 2% | 128 | 100% |
| 2 | 75 | 59% | 26 | 20% | 24 | 19% | 1 | 1% | 2 | 2% | 128 | 100% |
| 3 | 78 | 64% | 19 | 16% | 14 | 11% | 8 | 7% | 3 | 2% | 122 | 100% |
| 4 | 75 | 64% | 18 | 15% | 13 | 9% | 8 | 7% | 6 | 5% | 120 | 100% |
| 5 | 79 | 64% | 18 | 15% | 16 | 13% | 4 | 3% | 6 | 5% | 123 | 100% |
| 6 | 80 | 61% | 25 | 19% | 16 | 12% | 3 | 2% | 7 | 5% | 131 | 100% |
| 7 | 75 | 60% | 23 | 18% | 14 | 11% | 10 | 8% | 4 | 3% | 126 | 100% |
| 8 | 71 | 57% | 18 | 14% | 14 | 11% | 14 | 11% | 8 | 6% | 125 | 100% |
| 9 | 77 | 60% | 24 | 19% | 17 | 13% | 6 | 5% | 4 | 3% | 128 | 100% |
| 10 | 80 | 65% | 23 | 19% | 11 | 9% | 3 | 2% | 7 | 6% | 124 | 100% |
| 11 | 81 | 64% | 21 | 17% | 17 | 13% | 5 | 4% | 3 | 2% | 127 | 100% |
| 12 | 87 | 69% | 17 | 13% | 10 | 8% | 4 | 3% | 8 | 6% | 126 | 100% |
| 13 | 68 | 52% | 27 | 20% | 26 | 20% | 8 | 6% | 3 | 2% | 132 | 100% |
| 14 | 75 | 60% | 24 | 19% | 18 | 14% | 4 | 3% | 5 | 4% | 126 | 100% |
| 15 | 78 | 62% | 22 | 18% | 17 | 14% | 4 | 3% | 4 | 3% | 125 | 100% |
| 16 | 78 | 64% | 14 | 11% | 19 | 16% | 4 | 3% | 7 | 6% | 122 | 100% |
| Total | 1242 | 62% | 341 | 17% | 256 | 13% | 92 | 5% | 80 | 4% | 2013 | 100% |

|  | Value | df | p-value |
| --- | --- | --- | --- |
| Pearson Chi-Square | 69.153 | 60 | 0.19 |

6. Race:

|  | White | | African American | | Asian | | Hispanic | | Other | | Total | |
| --- | --- | --- | --- | --- | --- | --- | --- | --- | --- | --- | --- | --- |
|  | Count | % | Count | % | Count | % | Count | % | Count | % | Count | % |
| 1 | 91 | 71% | 13 | 10% | 3 | 2% | 14 | 11% | 7 | 5% | 128 | 100% |
| 2 | 87 | 68% | 21 | 16% | 8 | 6% | 9 | 7% | 3 | 2% | 128 | 100% |
| 3 | 88 | 72% | 15 | 12% | 10 | 8% | 5 | 4% | 4 | 3% | 122 | 100% |
| 4 | 92 | 78% | 11 | 9% | 7 | 6% | 7 | 6% | 3 | 3% | 120 | 100% |
| 5 | 86 | 70% | 12 | 10% | 11 | 9% | 7 | 6% | 7 | 6% | 123 | 100% |
| 6 | 103 | 79% | 10 | 8% | 12 | 9% | 3 | 2% | 3 | 2% | 131 | 100% |
| 7 | 92 | 73% | 16 | 13% | 4 | 3% | 12 | 10% | 2 | 2% | 126 | 100% |
| 8 | 84 | 67% | 16 | 13% | 11 | 9% | 7 | 6% | 7 | 6% | 125 | 100% |
| 9 | 108 | 84% | 9 | 7% | 2 | 2% | 3 | 2% | 6 | 5% | 128 | 100% |
| 10 | 88 | 71% | 10 | 8% | 12 | 10% | 9 | 7% | 5 | 4% | 124 | 100% |
| 11 | 97 | 76% | 14 | 11% | 9 | 7% | 5 | 4% | 2 | 2% | 127 | 100% |
| 12 | 92 | 73% | 14 | 11% | 5 | 4% | 14 | 11% | 1 | 1% | 126 | 100% |
| 13 | 102 | 77% | 18 | 14% | 4 | 3% | 5 | 4% | 3 | 2% | 132 | 100% |
| 14 | 96 | 76% | 12 | 10% | 9 | 7% | 4 | 3% | 5 | 4% | 126 | 100% |
| 15 | 97 | 78% | 14 | 11% | 7 | 6% | 6 | 5% | 1 | 1% | 125 | 100% |
| 16 | 95 | 78% | 10 | 8% | 5 | 4% | 9 | 7% | 3 | 2% | 122 | 100% |
| Total | 1498 | 74% | 215 | 11% | 119 | 6% | 117 | 6% | 62 | 3% | 2013 | 100% |

|  | Value | df | p-value |
| --- | --- | --- | --- |
| Pearson Chi-Square | 81.581 | 60 | 0.06 |

7. Health Status:

|  | Very poor | | Poor | | Fair | | Good | | Excellent | | Total | |
| --- | --- | --- | --- | --- | --- | --- | --- | --- | --- | --- | --- | --- |
|  | Count | % | Count | % | Count | % | Count | % | Count | % | Count | % |
| 1 | 0 | 0% | 8 | 6% | 24 | 19% | 65 | 51% | 31 | 24% | 128 | 100% |
| 2 | 0 | 0% | 4 | 3% | 25 | 20% | 77 | 60% | 22 | 17% | 128 | 100% |
| 3 | 0 | 0% | 4 | 3% | 28 | 23% | 65 | 53% | 25 | 20% | 122 | 100% |
| 4 | 1 | 1% | 5 | 4% | 25 | 21% | 63 | 53% | 26 | 22% | 120 | 100% |
| 5 | 1 | 1% | 4 | 3% | 25 | 20% | 64 | 52% | 29 | 24% | 123 | 100% |
| 6 | 1 | 1% | 5 | 4% | 17 | 13% | 74 | 56% | 34 | 26% | 131 | 100% |
| 7 | 1 | 1% | 3 | 2% | 24 | 19% | 59 | 47% | 39 | 31% | 126 | 100% |
| 8 | 0 | 0% | 3 | 2% | 20 | 16% | 67 | 54% | 35 | 28% | 125 | 100% |
| 9 | 0 | 0% | 8 | 6% | 21 | 16% | 71 | 55% | 28 | 22% | 128 | 100% |
| 10 | 0 | 0% | 3 | 2% | 26 | 21% | 70 | 56% | 25 | 20% | 124 | 100% |
| 11 | 1 | 1% | 2 | 2% | 25 | 20% | 69 | 54% | 30 | 24% | 127 | 100% |
| 12 | 1 | 1% | 3 | 2% | 20 | 16% | 71 | 56% | 31 | 25% | 126 | 100% |
| 13 | 0 | 0% | 5 | 4% | 35 | 27% | 59 | 45% | 33 | 25% | 132 | 100% |
| 14 | 0 | 0% | 4 | 3% | 24 | 19% | 74 | 59% | 24 | 19% | 126 | 100% |
| 15 | 0 | 0% | 4 | 3% | 20 | 16% | 70 | 56% | 31 | 25% | 125 | 100% |
| 16 | 1 | 1% | 2 | 2% | 22 | 18% | 72 | 59% | 25 | 20% | 122 | 100% |
| Total | 7 | 0% | 67 | 3% | 379 | 19% | 1090 | 54% | 468 | 23% | 2013 | 100% |

|  | Value | df | p-value |
| --- | --- | --- | --- |
| Pearson Chi-Square | 45.950 | 60 | 0.91 |

8. Computer experience:

|  | Extremely uncomfortable | | Somewhat uncomfortable | | Neither comfortable nor uncomfortable | | Somewhat comfortable | | Extremely comfortable | | Total | |
| --- | --- | --- | --- | --- | --- | --- | --- | --- | --- | --- | --- | --- |
|  | Count | % | Count | % | Count | % | Count | % | Count | % | Count | % |
| 1 | 2 | 2% | 1 | 1% | 3 | 2% | 29 | 23% | 93 | 73% | 128 | 100% |
| 2 | 0 | 0% | 1 | 1% | 3 | 2% | 25 | 20% | 99 | 77% | 128 | 100% |
| 3 | 0 | 0% | 2 | 2% | 5 | 4% | 29 | 24% | 86 | 70% | 122 | 100% |
| 4 | 1 | 1% | 2 | 1% | 4 | 3% | 29 | 25% | 84 | 71% | 120 | 100% |
| 5 | 0 | 0% | 0 | 0% | 4 | 3% | 27 | 22% | 92 | 75% | 123 | 100% |
| 6 | 1 | 1% | 1 | 1% | 7 | 5% | 20 | 15% | 102 | 78% | 131 | 100% |
| 7 | 0 | 0% | 1 | 1% | 2 | 2% | 22 | 17% | 101 | 80% | 126 | 100% |
| 8 | 0 | 0% | 0 | 0% | 0 | 0% | 24 | 19% | 101 | 81% | 125 | 100% |
| 9 | 1 | 1% | 2 | 2% | 1 | 1% | 23 | 18% | 101 | 79% | 128 | 100% |
| 10 | 1 | 1% | 0 | 0% | 3 | 2% | 27 | 22% | 93 | 75% | 124 | 100% |
| 11 | 0 | 0% | 1 | 1% | 2 | 2% | 28 | 22% | 96 | 76% | 127 | 100% |
| 12 | 1 | 1% | 2 | 2% | 3 | 2% | 30 | 24% | 90 | 71% | 126 | 100% |
| 13 | 0 | 0% | 1 | 1% | 5 | 4% | 25 | 19% | 101 | 77% | 132 | 100% |
| 14 | 0 | 0% | 0 | 0% | 1 | 1% | 22 | 17% | 103 | 82% | 126 | 100% |
| 15 | 1 | 1% | 1 | 1% | 0 | 0% | 23 | 18% | 100 | 80% | 125 | 100% |
| 16 | 1 | 1% | 1 | 1% | 7 | 6% | 24 | 20% | 89 | 73% | 122 | 100% |
| Total | 8 | 0% | 15 | 1% | 50 | 2% | 407 | 20% | 1531 | 76% | 2013 | 100% |

|  | Value | df | p-value |
| --- | --- | --- | --- |
| Pearson Chi-Square | 51.742 | 60 | 0.77 |
